# Supplementary material for: Impact of digital breast tomosynthesis on screening performance and interval cancer rates compared to digital mammography: A meta-analysis
Source: PLoS One. 2025 Jan 31;20(1):e0315466. doi: 10.1371/journal.pone.0315466 (PMC11785311; doi:10.1371/journal.pone.0315466)
Supplement: S1 File — (DOCX) [file pone.0315466.s001.docx]

**S1 File. Search strategies and procedures.**

**Keywords：**

Mammography; Mammographies; Digital Breast Tomosynthesis; Breast Tomosyntheses, Digital; Breast Tomosynthesis, Digital; Digital Breast Tomosyntheses; X-ray Breast Tomosynthesis; Breast Tomosyntheses, X-ray; Breast Tomosynthesis, X-ray; X-ray Breast Tomosyntheses; X ray Breast Tomosynthesis; 3D-Mammography; 3D-Mammographies; 3D Mammography; Digital Mammography; Digital Mammographies; Mammographies, Digital; Mammography, Digital; interval breast cancer

**PubMed:**

Search: (("Mammography"[Mesh]) OR ((((((((((((((((((Mammography[Title/Abstract]) OR (Mammographies[Title/Abstract])) OR (Digital Breast Tomosynthesis[Title/Abstract])) OR (Breast Tomosyntheses, Digital[Title/Abstract])) OR (Breast Tomosynthesis, Digital[Title/Abstract])) OR (Digital Breast Tomosyntheses[Title/Abstract])) OR (X-ray Breast Tomosynthesis[Title/Abstract])) OR (Breast Tomosyntheses, X-ray[Title/Abstract])) OR (Breast Tomosynthesis, X-ray[Title/Abstract])) OR (X-ray Breast Tomosyntheses[Title/Abstract])) OR (X ray Breast Tomosynthesis[Title/Abstract])) OR (3D-Mammography[Title/Abstract])) OR (3D Mammographies[Title/Abstract])) OR (3D Mammography[Title/Abstract])) OR (Digital Mammography[Title/Abstract])) OR (Digital Mammographies[Title/Abstract])) OR (Mammographies, Digital[Title/Abstract])) OR (Mammography, Digital[Title/Abstract]))) AND (interval breast cancer[Title/Abstract])

**Cochrane：**

#1 (Mammography):ab OR (X-ray Breast Tomosynthesis):ab OR (3D-Mammographies):ab OR (3D Mammography):ab OR (X-ray Breast Tomosyntheses):ab

#2 (Digital Breast Tomosynthesis):ab OR (Digital Breast Tomosyntheses):ab OR (X ray Breast Tomosynthesis):ab OR (Breast Tomosyntheses, Digital):ab OR (Breast Tomosynthesis, X-ray):ab

#3 (Breast Tomosynthesis, Digital):ab OR (Breast Tomosyntheses, X-ray):ab OR (3D-Mammography):ab OR (Mammographies):ab OR (Mammographies, Digital):ab

#4 (Digital Mammographies):ab OR (Digital Mammography):ab OR (Mammography, Digital):ab

#5 #1 OR #2 OR #3 OR #4

#6 interval breast cancer

#7 #5 AND #6

**Web of science:**

(TS=(Mammography) OR TS=(Mammographies) OR TS=(Digital Mammography) OR TS=(Digital Mammographies) OR TS=(Mammographies, Digital) OR TS=(Mammography, Digital) OR TS=(Digital Breast Tomosynthesis) OR TS=(Breast Tomosyntheses, Digital) OR TS=(Breast Tomosynthesis, Digital) OR TS=(Digital Breast Tomosyntheses) OR TS=(3D-Mammography) OR TS=(3D Mammography) OR TS=(3D-Mammographies) OR TS=(X-ray Breast Tomosynthesis) OR TS=(Breast Tomosyntheses, X-ray) OR TS=(Breast Tomosynthesis, X-ray) OR TS=(X ray Breast Tomosynthesis) OR TS=(X-ray Breast Tomosyntheses)) NOT (SILOID==("PPRN"))

AND

(TS=(interval breast cancer)) NOT (SILOID==("PPRN"))
