# Supplementary material for: Impact of digital breast tomosynthesis on screening performance and interval cancer rates compared to digital mammography: A meta-analysis
Source: PLoS One. 2025 Jan 31;20(1):e0315466. doi: 10.1371/journal.pone.0315466 (PMC11785311; doi:10.1371/journal.pone.0315466)
Supplement: S1 Table — (DOCX) [file pone.0315466.s003.docx]

**S1 Table. Study Characteristics and Patient Demographics**

| **Study (year)** | **Country** | **Study type** | **Age (mean or median)** | **Participants** | **Comparison** | **Interval cancer ascertainment**  **and follow-up (same for comparison?)** | **Period of screening**  **(same or different cohort)** | **Screen reading** | **RR(95% CI)** |
| --- | --- | --- | --- | --- | --- | --- | --- | --- | --- |
| Hofvind (2021) | Norway | Prospective | 59 | 28754 | DBT vs DM | Cancer Registry of Norway and population cancer registry;  2-year follow-up (yes) | Jan 2016 - Dec 2017  (same cohort) | Double reading with  consensus | 0.69(95% CI: 0.39, 1.22) |
| Hovda (2020) | Norway | Prospective | 59 | 92404 | SM/DBT vs DM | Cancer Registry of Norway and population cancer registry;  2-year follow-up (yes) | SM/DBT: Feb 2014- Dec 2015  DM: Feb 2016 - Dec 2017  (same cohort) | Double reading with  consensus | 1.29(95% CI: 0.94, 1.77) |
| Pattacini (2022) | Italy | Prospective | 55 | 26877 | DM/DBT vs DM | Cancer registry and pathology 2-year follow-up (yes) | Mar 2014 - Aug 2017  (same cohort) | Double reading plus arbitration | 0.97(95% CI: 0.53, 1.8) |
| Pulido-Carmona  (2024) | Spain | Prospective | 58 | 39913 | DM/DBT vs DM | Cancer Registry of Córdoba and medical database;  2-year follow-up (yes) | Jan 2015 - Dec 2016  (same cohort) | Double reading | 0.51(95% CI: 0.28, 0.93) |
| Armaroli (2022) | Italy | Prospective | 57 | 73866 | DBT vs DM | NR; 3-year follow-up (unclear) | Dec 2014 - Dec 2017  (same cohort) | Double reading | 0.92(95% CI: 1.01, 1.31) |
| Bernardi (2021) | Italy | Prospective | 58 | 83779 | SM/DBT vs DM | Cancer Registry, pathology and hospital databases;  2-year follow-up (unclear) | SM/DBT: Oct 2014 - Oct 2016  DM: Jan 2013 - Oct 2014  (same cohort) | Double reading plus arbitration | 0.81(95% CI: 1.34, 1.87) |
| Skaane (2018) | Norway | Prospective | 59 | 84175 | DM/DBT vs DM | Cancer Registry of Norway and  population cancer registry; 2-year follow-up (yes) | DM/DBT: Nov 2010 - Dec 2012  DM: 2006 - 2009  (same cohort) | Double reading with  consensus | NR |
| McDonald (2016) | USA | Retrospective | 57 | 44468 | DM/DBT vs DM | Cancer Registry and hospital databases;  2-year follow-up (yes) | DM: 2010 - 2011  DM/DBT: 2011 - 2014  (same cohort) | Double reading with  consensus | 0.71(95% CI: 0.50, 1.00) |
| Hovda (2019) | Norway | Retrospective | 60 | 19082 | DM/DBT vs DM | Pathology and hospital databases; 2-year follow-up (yes) | Aug 2011 - Jun 2016  (same cohort) | Double reading with  consensus | NR |
| Houssami (2018) | Italy | Prospective | 58 | 32350 | DM/DBT vs DM | Cancer Registry of Norway and population cancer registry;  2-year follow-up (yes) | DM/DBT: Oct 2014 - Oct 2016  DM: Jan 2013 - Oct 2014  (same cohort) | Double reading | NR |
| Winter (2020) | USA | Retrospective | 60 | 236845 | DBT vs DM | Hospital databases;  1-year follow-up (yes) | DM: Jan 2012 - Dec 2014  DBT: Jan 2016 - Dec 2018  (same cohort) | NR | NR |

NR= not reported.

DBT=digital breast tomosynthesis (DBT) alone, DM/DBT=digital mammography (DM) plus DBT, SM/DBT= synthesized mammography (SM) plus DBT. RR (risk ratio) = the risk of interval cancer among women screened with DBT or combined with DBT imaging, compared to women screened with DM alone.
