# Supplementary material for: Impact of digital breast tomosynthesis on screening performance and interval cancer rates compared to digital mammography: A meta-analysis
Source: PLoS One. 2025 Jan 31;20(1):e0315466. doi: 10.1371/journal.pone.0315466 (PMC11785311; doi:10.1371/journal.pone.0315466)
Supplement: S2 Table — (DOCX) [file pone.0315466.s004.docx]

**S2** **Table. Meta-regression analysis.**

| Parameter | category | studies | Sensitivity(95%CI) | P1 | Specificity(95%CI) | P2 |
| --- | --- | --- | --- | --- | --- | --- |
| Iindex test | Yes | 8 | 85% (80-90) | P<0.001 | 97% (96-98) | P<0.001 |
|  | No | 3 | 89% (83-95) | . | 95% (91-98) | . |
| type | Yes | 8 | 85% (80-90) | P<0.001 | 97% (96-98) | P<0.001 |
|  | No | 3 | 88% (81-95) | . | 95% (92-98) | . |

index test=DBT alone or in combination with DBT；type= Prospective or Retrospective.
