# Supplementary material for: Impact of digital breast tomosynthesis on screening performance and interval cancer rates compared to digital mammography: A meta-analysis
Source: PLoS One. 2025 Jan 31;20(1):e0315466. doi: 10.1371/journal.pone.0315466 (PMC11785311; doi:10.1371/journal.pone.0315466)
Supplement: S3 Table — (DOCX) [file pone.0315466.s005.docx]

| **ALL ARTICLES** | | | |
| --- | --- | --- | --- |
| **Study** | | | **Reasons for inclusion or exclusion** |
| Moshina N | 2024 | ([1](#_ENREF_1)) | Results do not apply |
| Giorgi Rossi P | 2024 | ([2](#_ENREF_2)) | Results do not apply |
| Majid SZ | 2024 | ([3](#_ENREF_3)) | Results do not apply |
| Orsini L | 2024 | ([4](#_ENREF_4)) | Not comparative study |
| Nykanen A | 2024 | ([5](#_ENREF_5)) | Not comparative study |
| Mullen LA | 2024 | ([6](#_ENREF_6)) | Not comparative study |
| Celik L | 2024 | ([7](#_ENREF_7)) | Not comparative study |
| ten Velde DE | 2024 | ([8](#_ENREF_8)) | Not comparative study |
| Song H | 2024 | ([9](#_ENREF_9)) | Not comparative study |
| Sprague BL | 2023 | ([10](#_ENREF_10)) | Results do not apply |
| Skaane P | 2023 | ([11](#_ENREF_11)) | Results do not apply |
| Shima A | 2023 | ([12](#_ENREF_12)) | Not comparative study |
| Ramadas K | 2023 | ([13](#_ENREF_13)) | Not comparative study |
| Larsen M | 2023 | ([14](#_ENREF_14)) | Not comparative study |
| Lång K | 2023 | ([15](#_ENREF_15)) | Not comparative study |
| Vachon CM | 2023 | ([16](#_ENREF_16)) | Not comparative study |
| Holen Å | 2023 | ([17](#_ENREF_17)) | Not comparative study |
| Heinze F | 2023 | ([18](#_ENREF_18)) | Not comparative study |
| Moger TA | 2022 | ([19](#_ENREF_19)) | Results do not apply |
| Kerlikowske K | 2022 | ([20](#_ENREF_20)) | Results do not apply |
| Heindel W | 2022 | ([21](#_ENREF_21)) | Results do not apply |
| Heindel W | 2022 | ([22](#_ENREF_22)) | Results do not apply |
| Zhang Z | 2022 | ([23](#_ENREF_23)) | Not comparative study |
| Zhang Z | 2022 | ([24](#_ENREF_24)) | Not comparative study |
| Wanders AJT | 2022 | ([25](#_ENREF_25)) | Not comparative study |
| Ugalde-Morales E | 2022 | ([26](#_ENREF_26)) | Not comparative study |
| Timmermans L | 2022 | ([27](#_ENREF_27)) | Not comparative study |
| Seely JM | 2022 | ([28](#_ENREF_28)) | Not comparative study |
| Ryser MD | 2022 | ([29](#_ENREF_29)) | Not comparative study |
| Noguchi N | 2022 | ([30](#_ENREF_30)) | Not comparative study |
| Hovda T | 2022 | ([31](#_ENREF_31)) | Not comparative study |
| Friedewald SM | 2022 | ([32](#_ENREF_32)) | Not comparative study |
| Byng D | 2022 | ([33](#_ENREF_33)) | Not comparative study |
| Martiniussen MA | 2022 | ([34](#_ENREF_34)) | Not comparative study |
| Johnson K | 2021 | ([35](#_ENREF_35)) | Results do not apply |
| Zhu X | 2021 | ([36](#_ENREF_36)) | Not comparative study |
| Taourel P | 2021 | ([37](#_ENREF_37)) | Comment |
| Nguyen TL | 2021 | ([38](#_ENREF_38)) | Not comparative study |
| Lang K | 2021 | ([39](#_ENREF_39)) | Not comparative study |
| Harada-Shoji N | 2021 | ([40](#_ENREF_40)) | Not comparative study |
| Graewingholt A | 2021 | ([41](#_ENREF_41)) | Not comparative study |
| van Zelst JC | 2020 | ([42](#_ENREF_42)) | Not comparative study |
| Shieh Y | 2020 | ([43](#_ENREF_43)) | Not comparative study |
| Pisano E | 2020 | ([44](#_ENREF_44)) | Not comparative study |
| Njor SH | 2020 | ([45](#_ENREF_45)) | Not comparative study |
| Niraula S | 2020 | ([46](#_ENREF_46)) | Not comparative study |
| Nguyen TL | 2020 | ([47](#_ENREF_47)) | Not comparative study |
| Luker GD | 2020 | ([48](#_ENREF_48)) | Not comparative study |
| Comstock CE | 2020 | ([49](#_ENREF_49)) | Not comparative study |
| Román M | 2019 | ([50](#_ENREF_50)) | Results do not apply |
| Pilewskie M | 2019 | ([51](#_ENREF_51)) | Results do not apply |
| Hofvind S | 2019 | ([52](#_ENREF_52)) | Results do not apply |
| Tilanus-Linthorst MM | 2019 | ([53](#_ENREF_53)) | Not comparative study |
| Steponaviciene L | 2019 | ([54](#_ENREF_54)) | Not comparative study |
| Weigel S | 2018 | ([55](#_ENREF_55)) | Results do not apply |
| Pattacini P | 2018 | ([56](#_ENREF_56)) | Results do not apply |
| Bahl M | 2018 | ([57](#_ENREF_57)) | Results do not apply |
| Hofvind S | 2018 | ([58](#_ENREF_58)) | Not comparative study |
| Sayed S | 2023 | ([59](#_ENREF_59)) | Review |
| Glechner A | 2023 | ([60](#_ENREF_60)) | Review |
| Zeng A | 2022 | ([61](#_ENREF_61)) | Review |
| Hovda T | 2022 | ([62](#_ENREF_62)) | Review |
| Farber R | 2022 | ([63](#_ENREF_63)) | Review |
| Tsuruda KM | 2021 | ([64](#_ENREF_64)) | Review |
| Mullooly M | 2021 | ([65](#_ENREF_65)) | Review |
| Houssami N | 2021 | ([66](#_ENREF_66)) | Review |
| Houssami N | 2021 | ([67](#_ENREF_67)) | Review |
| Messinger J | 2019 | ([68](#_ENREF_68)) | Review |
| Mandoul C | 2019 | ([69](#_ENREF_69)) | Review |
| Destounis S | 2018 | ([70](#_ENREF_70)) | Review |
| Lekanidi K | 2017 | ([71](#_ENREF_71)) | Review |
| Houssami N | 2017 | ([72](#_ENREF_72)) | Review |
| Moschetti I | 2016 | ([73](#_ENREF_73)) | Review |
| Abdel‐Aleem H | 2016 | ([74](#_ENREF_74)) | Review |
| Knox M | 2015 | ([75](#_ENREF_75)) | Review |
| Payne JI | 2013 | ([76](#_ENREF_76)) | Review |
| Gøtzsche PC | 2013 | ([77](#_ENREF_77)) | Review |
| Goodwin A | 2013 | ([78](#_ENREF_78)) | Review |
| Edwards AGK | 2013 | ([79](#_ENREF_79)) | Review |
| Hoff SR | 2012 | ([80](#_ENREF_80)) | Review |
| Pellegrini M | 2011 | ([81](#_ENREF_81)) | Review |
| Ciatto S | 2011 | ([82](#_ENREF_82)) | Review |
| Bennett ML | 2011 | ([83](#_ENREF_83)) | Review |
| Magnus MC | 2011 | ([84](#_ENREF_84)) | Review |
| Elena PM | 2009 | ([85](#_ENREF_85)) | Review |
| Nothacker M | 2009 | ([86](#_ENREF_86)) | Review |
| Miller D | 2008 | ([87](#_ENREF_87)) | Review |
| Dale J | 2008 | ([88](#_ENREF_88)) | Review |
| Gordon PB | 2007 | ([89](#_ENREF_89)) | Review |
| Houssami N | 2006 | ([90](#_ENREF_90)) | Review |
| Hofvind S | 2005 | ([91](#_ENREF_91)) | Review |
| Baker S | 2005 | ([92](#_ENREF_92)) | Review |
| Ratner PA | 2001 | ([93](#_ENREF_93)) | Review |
| Bonfill Cosp X | 2001 | ([94](#_ENREF_94)) | Review |
| Banks E | 2001 | ([95](#_ENREF_95)) | Review |
| de Rijke JM | 2000 | ([96](#_ENREF_96)) | Review |
| Tosteson TD | 1999 | ([97](#_ENREF_97)) | Review |
| Koivunen D | 1994 | ([98](#_ENREF_98)) | Review |
| Ray KM | 2024 | ([99](#_ENREF_99)) | Review |
| Andersen SB | 2014 | ([100](#_ENREF_100)) | Review |
| Summaries for Patients | 2015 | ([101](#_ENREF_101)) | Comment |
| Acosta-Benito MA | 2016 | ([102](#_ENREF_102)) | Report |
| Liston JC | 1997 | ([103](#_ENREF_103)) | Report |
| Sakurai K | 2015 | ([104](#_ENREF_104)) | Report |
| Kaplan HG | 2019 | ([105](#_ENREF_105)) | Meeting |
| Singh D | 2018 | ([106](#_ENREF_106)) | Meeting |
| Ahn S | 2018 | ([107](#_ENREF_107)) | Meeting |
| Czene K | 2017 | ([108](#_ENREF_108)) | Meeting |
| Ferguson J | 2016 | ([109](#_ENREF_109)) | Meeting |
| De Munck L | 2016 | ([110](#_ENREF_110)) | Meeting |
| Sakurai K | 2015 | ([104](#_ENREF_104)) | Meeting |
| Jeitler K | 2015 | ([111](#_ENREF_111)) | Meeting |
| Klingen TA | 2013 | ([112](#_ENREF_112)) | Meeting |
| Andersen SB | 2013 | ([113](#_ENREF_113)) | Meeting |
| Sala M | 2011 | ([114](#_ENREF_114)) | Meeting |
| Musolino A | 2010 | ([115](#_ENREF_115)) | Meeting |
| Rayson D | 2009 | ([116](#_ENREF_116)) | Meeting |
| Maygarden SJ | 2006 | ([117](#_ENREF_117)) | Meeting |
| Huang C | 2010 | ([118](#_ENREF_118)) | Meeting |
| Banik S | 2009 | ([119](#_ENREF_119)) | Meeting |
| Chlebowski RT | 2007 | ([120](#_ENREF_120)) | Meeting |
| Mills C | 2024 | ([121](#_ENREF_121)) | Inconsistent with the theme |
| Hubbard RA | 2024 | ([122](#_ENREF_122)) | Inconsistent with the theme |
| Freitas-Junior R | 2024 | ([123](#_ENREF_123)) | Inconsistent with the theme |
| Dunn J | 2024 | ([124](#_ENREF_124)) | Inconsistent with the theme |
| de Munck L | 2024 | ([125](#_ENREF_125)) | Inconsistent with the theme |
| Ye Z | 2023 | ([126](#_ENREF_126)) | Inconsistent with the theme |
| Sendur HN | 2023 | ([127](#_ENREF_127)) | Comment |
| Razavi M | 2023 | ([128](#_ENREF_128)) | Inconsistent with the theme |
| Rahim A | 2023 | ([129](#_ENREF_129)) | Inconsistent with the theme |
| Lee JM | 2023 | ([130](#_ENREF_130)) | Inconsistent with the theme |
| Kou K | 2023 | ([131](#_ENREF_131)) | Inconsistent with the theme |
| Khodayarian M | 2023 | ([132](#_ENREF_132)) | Inconsistent with the theme |
| Han HJ | 2023 | ([133](#_ENREF_133)) | Inconsistent with the theme |
| Grigg J | 2023 | ([134](#_ENREF_134)) | Inconsistent with the theme |
| Goossens MM | 2023 | ([135](#_ENREF_135)) | Inconsistent with the theme |
| Freitas V | 2023 | ([136](#_ENREF_136)) | Inconsistent with the theme |
| Fitzpatrick P | 2023 | ([137](#_ENREF_137)) | Inconsistent with the theme |
| Eijkelboom AH | 2023 | ([138](#_ENREF_138)) | Inconsistent with the theme |
| Claringbold L | 2023 | ([139](#_ENREF_139)) | Inconsistent with the theme |
| Çelik L | 2023 | ([140](#_ENREF_140)) | Inconsistent with the theme |
| Ambinder EB | 2023 | ([141](#_ENREF_141)) | Inconsistent with the theme |
| Hacek RT | 2022 | ([142](#_ENREF_142)) | Inconsistent with the theme |
| Groome PA | 2022 | ([143](#_ENREF_143)) | Inconsistent with the theme |
| Gordon PB | 2022 | ([144](#_ENREF_144)) | Inconsistent with the theme |
| Dreher N | 2022 | ([145](#_ENREF_145)) | Inconsistent with the theme |
| Bucchi L | 2022 | ([146](#_ENREF_146)) | Inconsistent with the theme |
| Boyle T | 2022 | ([147](#_ENREF_147)) | Inconsistent with the theme |
| Blackmore KM | 2022 | ([148](#_ENREF_148)) | Inconsistent with the theme |
| Bertrand C | 2022 | ([149](#_ENREF_149)) | Inconsistent with the theme |
| Alabousi A | 2022 | ([150](#_ENREF_150)) | Inconsistent with the theme |
| Yu H | 2021 | ([151](#_ENREF_151)) | Inconsistent with the theme |
| Wang S | 2021 | ([152](#_ENREF_152)) | Inconsistent with the theme |
| van Ravesteyn NT | 2021 | ([153](#_ENREF_153)) | Inconsistent with the theme |
| Tsuruda KM | 2021 | ([154](#_ENREF_154)) | Inconsistent with the theme |
| Sardu C | 2021 | ([155](#_ENREF_155)) | Inconsistent with the theme |
| Park VY | 2021 | ([156](#_ENREF_156)) | Inconsistent with the theme |
| Monib S | 2021 | ([157](#_ENREF_157)) | Inconsistent with the theme |
| McWilliams L | 2021 | ([158](#_ENREF_158)) | Inconsistent with the theme |
| Mao Z | 2021 | ([159](#_ENREF_159)) | Inconsistent with the theme |
| Mao N | 2021 | ([160](#_ENREF_160)) | Inconsistent with the theme |
| Mango VL | 2021 | ([161](#_ENREF_161)) | Inconsistent with the theme |
| Lynch T | 2021 | ([162](#_ENREF_162)) | Inconsistent with the theme |
| Kim GR | 2021 | ([163](#_ENREF_163)) | Inconsistent with the theme |
| Hersch J | 2021 | ([164](#_ENREF_164)) | Inconsistent with the theme |
| Frankhauser DE | 2021 | ([165](#_ENREF_165)) | Inconsistent with the theme |
| Daniaux M | 2021 | ([166](#_ENREF_166)) | Inconsistent with the theme |
| Chang JM | 2021 | ([167](#_ENREF_167)) | Inconsistent with the theme |
| Burnside ES | 2021 | ([168](#_ENREF_168)) | Inconsistent with the theme |
| Bucchi L | 2021 | ([169](#_ENREF_169)) | Inconsistent with the theme |
| Aghamohammadi V | 2021 | ([170](#_ENREF_170)) | Inconsistent with the theme |
| MacInnes EG | 2020 | ([171](#_ENREF_171)) | Inconsistent with the theme |
| Liu Q | 2020 | ([172](#_ENREF_172)) | Inconsistent with the theme |
| Kaplan HG | 2020 | ([173](#_ENREF_173)) | Inconsistent with the theme |
| Irvin VL | 2020 | ([174](#_ENREF_174)) | Inconsistent with the theme |
| Hudson SM | 2020 | ([175](#_ENREF_175)) | Inconsistent with the theme |
| Holen Å | 2020 | ([176](#_ENREF_176)) | Inconsistent with the theme |
| Henderson LM | 2020 | ([177](#_ENREF_177)) | Inconsistent with the theme |
| Duffy S | 2020 | ([178](#_ENREF_178)) | Inconsistent with the theme |
| Costanza ME | 2020 | ([179](#_ENREF_179)) | Inconsistent with the theme |
| Chlebowski RT | 2020 | ([180](#_ENREF_180)) | Inconsistent with the theme |
| Cabioglu N | 2020 | ([181](#_ENREF_181)) | Inconsistent with the theme |
| Barros AF | 2020 | ([182](#_ENREF_182)) | Inconsistent with the theme |
| Azam S | 2020 | ([183](#_ENREF_183)) | Inconsistent with the theme |
| Abdolell M | 2020 | ([184](#_ENREF_184)) | Inconsistent with the theme |
| Yang GE | 2019 | ([185](#_ENREF_185)) | Inconsistent with the theme |
| Tabár L | 2019 | ([186](#_ENREF_186)) | Inconsistent with the theme |
| Strand F | 2019 | ([187](#_ENREF_187)) | Inconsistent with the theme |
| Rannikko A | 2019 | ([188](#_ENREF_188)) | Inconsistent with the theme |
| Prange A | 2019 | ([189](#_ENREF_189)) | Inconsistent with the theme |
| Mannu GS | 2019 | ([190](#_ENREF_190)) | Inconsistent with the theme |
| Lee Y | 2019 | ([191](#_ENREF_191)) | Inconsistent with the theme |
| Lee JS | 2019 | ([192](#_ENREF_192)) | Inconsistent with the theme |
| Kerlikowske K | 2019 | ([193](#_ENREF_193)) | Inconsistent with the theme |
| Iotti V | 2019 | ([194](#_ENREF_194)) | Inconsistent with the theme |
| Ho PJ | 2019 | ([195](#_ENREF_195)) | Inconsistent with the theme |
| Hinton B | 2019 | ([196](#_ENREF_196)) | Inconsistent with the theme |
| Hinton B | 2019 | ([197](#_ENREF_197)) | Inconsistent with the theme |
| Haas JS | 2019 | ([198](#_ENREF_198)) | Inconsistent with the theme |
| Grassmann F | 2019 | ([199](#_ENREF_199)) | Inconsistent with the theme |
| Cheasley D | 2019 | ([200](#_ENREF_200)) | Inconsistent with the theme |
| Bhargava S | 2019 | ([201](#_ENREF_201)) | Inconsistent with the theme |
| Bakker MF | 2019 | ([202](#_ENREF_202)) | Inconsistent with the theme |
| Aarts AMWM | 2019 | ([203](#_ENREF_203)) | Inconsistent with the theme |
| You JK | 2018 | ([204](#_ENREF_204)) | Inconsistent with the theme |
| van Bommel RMG | 2018 | ([205](#_ENREF_205)) | Inconsistent with the theme |
| van Bommel R | 2018 | ([206](#_ENREF_206)) | Inconsistent with the theme |
| Unger-Saldana K | 2018 | ([207](#_ENREF_207)) | Inconsistent with the theme |
| Strand F | 2018 | ([208](#_ENREF_208)) | Inconsistent with the theme  (dissertation thesis) |
| Slater JS | 2018 | ([209](#_ENREF_209)) | Inconsistent with the theme |
| Singh D | 2018 | ([210](#_ENREF_210)) | Inconsistent with the theme |
| Sankatsing VDV | 2018 | ([211](#_ENREF_211)) | Inconsistent with the theme |
| Sala M | 2018 | ([212](#_ENREF_212)) | Inconsistent with the theme |
| Puvanesarajah S | 2018 | ([213](#_ENREF_213)) | Inconsistent with the theme |
| Park VY | 2018 | ([214](#_ENREF_214)) | Inconsistent with the theme |
| Nguyen TL | 2018 | ([215](#_ENREF_215)) | Inconsistent with the theme |
| Murphy DR | 2018 | ([216](#_ENREF_216)) | Inconsistent with the theme |
| Moshina N | 2018 | ([217](#_ENREF_217)) | Inconsistent with the theme |
| Moon HJ | 2018 | ([218](#_ENREF_218)) | Inconsistent with the theme |
| Mohd Mujar NM | 2018 | ([219](#_ENREF_219)) | Inconsistent with the theme |
| Mireles-Aguilar T | 2018 | ([220](#_ENREF_220)) | Inconsistent with the theme |
| Lee JM | 2018 | ([221](#_ENREF_221)) | Inconsistent with the theme |
| Kerlikowske K | 2018 | ([222](#_ENREF_222)) | Inconsistent with the theme |
| Jodal HC | 2018 | ([223](#_ENREF_223)) | Inconsistent with the theme |
| Jiang L | 2018 | ([224](#_ENREF_224)) | Inconsistent with the theme |
| Hinton BJ | 2018 | ([225](#_ENREF_225)) | Inconsistent with the theme  (dissertation thesis) |
| Defossez G | 2018 | ([226](#_ENREF_226)) | Inconsistent with the theme |
| Chan EK | 2018 | ([227](#_ENREF_227)) | Inconsistent with the theme |
| Cardoso de Almeida T | 2018 | ([228](#_ENREF_228)) | Inconsistent with the theme |
| Burnside ES | 2018 | ([229](#_ENREF_229)) | Inconsistent with the theme |
| Brück N | 2018 | ([230](#_ENREF_230)) | Inconsistent with the theme |
| Yen AM-F | 2017 | ([231](#_ENREF_231)) | Inconsistent with the theme |
| Wernli KJ | 2017 | ([232](#_ENREF_232)) | Inconsistent with the theme |
| Wanders JOP | 2017 | ([233](#_ENREF_233)) | Inconsistent with the theme |
| van Bommel RMG | 2017 | ([234](#_ENREF_234)) | Inconsistent with the theme |
| Tohno E | 2017 | ([235](#_ENREF_235)) | Inconsistent with the theme |
| Timmermans L | 2017 | ([236](#_ENREF_236)) | Inconsistent with the theme |
| Strand F | 2017 | ([237](#_ENREF_237)) | Inconsistent with the theme |
| Scoggins M | 2017 | ([238](#_ENREF_238)) | Inconsistent with the theme |
| Román M | 2017 | ([239](#_ENREF_239)) | Inconsistent with the theme |
| Rauscher GH | 2017 | ([240](#_ENREF_240)) | Inconsistent with the theme |
| Ott OJ | 2017 | ([241](#_ENREF_241)) | Inconsistent with the theme |
| Moshina N | 2017 | ([242](#_ENREF_242)) | Inconsistent with the theme |
| Miglioretti DL | 2017 | ([243](#_ENREF_243)) | Inconsistent with the theme |
| Mennella S | 2017 | ([244](#_ENREF_244)) | Inconsistent with the theme |
| Lekanidi K | 2017 | ([245](#_ENREF_245)) | Inconsistent with the theme |
| Houssami N | 2017 | ([246](#_ENREF_246)) | Inconsistent with the theme |
| Holt RE | 2017 | ([247](#_ENREF_247)) | Inconsistent with the theme |
| Delacour-Billon S | 2017 | ([248](#_ENREF_248)) | Inconsistent with the theme |
| Bellio G | 2017 | ([249](#_ENREF_249)) | Inconsistent with the theme |
| Arleo EK | 2017 | ([250](#_ENREF_250)) | Inconsistent with the theme |
| Youl PH | 2016 | ([251](#_ENREF_251)) | Inconsistent with the theme |
| Weber RJP | 2016 | ([252](#_ENREF_252)) | Inconsistent with the theme |
| Weber RJP | 2016 | ([253](#_ENREF_253)) | Inconsistent with the theme |
| Trentham-Dietz A | 2016 | ([254](#_ENREF_254)) | Inconsistent with the theme |
| Strand F | 2016 | ([255](#_ENREF_255)) | Inconsistent with the theme |
| Sripaiboonkij N | 2016 | ([256](#_ENREF_256)) | Inconsistent with the theme |
| Shieh Y | 2016 | ([257](#_ENREF_257)) | Inconsistent with the theme |
| Salvagnini E | 2016 | ([258](#_ENREF_258)) | Inconsistent with the theme |
| Pepe MS | 2016 | ([259](#_ENREF_259)) | Inconsistent with the theme |
| Ohuchi N | 2016 | ([260](#_ENREF_260)) | Inconsistent with the theme |
| Oeffinger KC | 2016 | ([261](#_ENREF_261)) | Inconsistent with the theme |
| Massat NJ | 2016 | ([262](#_ENREF_262)) | Inconsistent with the theme |
| Marshall JK | 2016 | ([263](#_ENREF_263)) | Inconsistent with the theme |
| Lee K | 2016 | ([264](#_ENREF_264)) | Inconsistent with the theme |
| Hsieh JC-F | 2016 | ([265](#_ENREF_265)) | Inconsistent with the theme |
| Houssami N | 2016 | ([266](#_ENREF_266)) | Inconsistent with the theme |
| Goodrich ME | 2016 | ([267](#_ENREF_267)) | Inconsistent with the theme |
| Duffy SW | 2016 | ([268](#_ENREF_268)) | Inconsistent with the theme |
| Choi WJ | 2016 | ([269](#_ENREF_269)) | Inconsistent with the theme |
| Best AL | 2016 | ([270](#_ENREF_270)) | Inconsistent with the theme |
| Berg CD | 2016 | ([271](#_ENREF_271)) | Inconsistent with the theme |
| O'Mahony JF | 2015 | ([272](#_ENREF_272)) | Inconsistent with the theme |
| O'Brien KM | 2015 | ([273](#_ENREF_273)) | Inconsistent with the theme |
| Meshkat B | 2015 | ([274](#_ENREF_274)) | Inconsistent with the theme |
| Li J | 2015 | ([275](#_ENREF_275)) | Inconsistent with the theme |
| Lee JM | 2015 | ([276](#_ENREF_276)) | Inconsistent with the theme |
| Knox M | 2015 | ([277](#_ENREF_277)) | Inconsistent with the theme |
| Kerlikowske K | 2015 | ([278](#_ENREF_278)) | Inconsistent with the theme |
| Jones BA | 2015 | ([279](#_ENREF_279)) | Inconsistent with the theme |
| Johnson JM | 2015 | ([280](#_ENREF_280)) | Inconsistent with the theme |
| Holm J | 2015 | ([281](#_ENREF_281)) | Inconsistent with the theme |
| Henderson LM | 2015 | ([282](#_ENREF_282)) | Inconsistent with the theme |
| Gummersbach E | 2015 | ([283](#_ENREF_283)) | Inconsistent with the theme |
| Emaus MJ | 2015 | ([284](#_ENREF_284)) | Inconsistent with the theme |
| Bucchi L | 2015 | ([285](#_ENREF_285)) | Inconsistent with the theme |
| Bare M | 2015 | ([286](#_ENREF_286)) | Inconsistent with the theme |
| Andersen SB | 2015 | ([287](#_ENREF_287)) | Inconsistent with the theme |
| Urbschat I | 2014 | ([288](#_ENREF_288)) | Inconsistent with the theme |
| Taylor-Phillips S | 2014 | ([289](#_ENREF_289)) | Inconsistent with the theme |
| Simon MS | 2014 | ([290](#_ENREF_290)) | Inconsistent with the theme |
| Seetoh T | 2014 | ([291](#_ENREF_291)) | Inconsistent with the theme |
| Renart-Vicens G | 2014 | ([292](#_ENREF_292)) | Inconsistent with the theme |
| Nederend J | 2014 | ([293](#_ENREF_293)) | Inconsistent with the theme |
| Nederend J | 2014 | ([294](#_ENREF_294)) | Inconsistent with the theme |
| Narod SA | 2014 | ([295](#_ENREF_295)) | Inconsistent with the theme |
| Miller AB | 2014 | ([296](#_ENREF_296)) | Inconsistent with the theme |
| Kawai M | 2014 | ([297](#_ENREF_297)) | Inconsistent with the theme |
| Ishida T | 2014 | ([298](#_ENREF_298)) | Inconsistent with the theme |
| Hofvind S | 2014 | ([299](#_ENREF_299)) | Inconsistent with the theme |
| Fontein DB | 2014 | ([300](#_ENREF_300)) | Inconsistent with the theme |
| Fong Y | 2014 | ([301](#_ENREF_301)) | Inconsistent with the theme |
| Domingo L | 2014 | ([302](#_ENREF_302)) | Inconsistent with the theme |
| Dibden A | 2014 | ([303](#_ENREF_303)) | Inconsistent with the theme |
| Carbonaro LA | 2014 | ([304](#_ENREF_304)) | Inconsistent with the theme |
| Boyd NF | 2014 | ([305](#_ENREF_305)) | Inconsistent with the theme |
| Blanch J | 2014 | ([306](#_ENREF_306)) | Inconsistent with the theme |
| Bento MJ | 2014 | ([307](#_ENREF_307)) | Inconsistent with the theme |
| Autier P | 2014 | ([308](#_ENREF_308)) | Inconsistent with the theme |
| Araujo MC | 2014 | ([309](#_ENREF_309)) | Inconsistent with the theme |
| Rafferty EA | 2013 | ([310](#_ENREF_310)) | Inconsistent with the theme |
| Prieto Garcia MA | 2013 | ([311](#_ENREF_311)) | Inconsistent with the theme |
| Paci E | 2013 | ([312](#_ENREF_312)) | Inconsistent with the theme |
| Houssami N | 2013 | ([313](#_ENREF_313)) | Inconsistent with the theme |
| Hoff SR | 2013 | ([314](#_ENREF_314)) | Correction |
| Eriksson L | 2013 | ([315](#_ENREF_315)) | Inconsistent with the theme |
| Duffy SW | 2013 | ([316](#_ENREF_316)) | Inconsistent with the theme |
| Domingo L | 2013 | ([317](#_ENREF_317)) | Inconsistent with the theme |
| Dittus K | 2013 | ([318](#_ENREF_318)) | Inconsistent with the theme |
| De Koning H | 2013 | ([319](#_ENREF_319)) | Inconsistent with the theme |
| Cappello NM | 2013 | ([320](#_ENREF_320)) | Inconsistent with the theme |
| Wang JH | 2012 | ([321](#_ENREF_321)) | Inconsistent with the theme |
| Speiser D | 2012 | ([322](#_ENREF_322)) | Inconsistent with the theme |
| Solbjør M | 2012 | ([323](#_ENREF_323)) | Inconsistent with the theme |
| Solbjor M | 2012 | ([324](#_ENREF_324)) | Inconsistent with the theme |
| Peplonska B | 2012 | ([325](#_ENREF_325)) | Inconsistent with the theme |
| Pagán JA | 2012 | ([326](#_ENREF_326)) | Inconsistent with the theme |
| McLaughlin JM | 2012 | ([327](#_ENREF_327)) | Inconsistent with the theme |
| Lin C | 2012 | ([328](#_ENREF_328)) | Inconsistent with the theme |
| Kalager M | 2012 | ([329](#_ENREF_329)) | Inconsistent with the theme |
| Ishikawa Y | 2012 | ([330](#_ENREF_330)) | Inconsistent with the theme |
| Hymas RV | 2012 | ([331](#_ENREF_331)) | Inconsistent with the theme |
| Heidinger O | 2012 | ([332](#_ENREF_332)) | Inconsistent with the theme |
| Emaus M | 2012 | ([333](#_ENREF_333)) | Inconsistent with the theme |
| Crandall CJ | 2012 | ([334](#_ENREF_334)) | Inconsistent with the theme |
| Carney PA | 2012 | ([335](#_ENREF_335)) | Inconsistent with the theme |
| Berg WA | 2012 | ([336](#_ENREF_336)) | Inconsistent with the theme |
| Rayson D | 2011 | ([337](#_ENREF_337)) | Inconsistent with the theme |
| Parvinen I | 2011 | ([338](#_ENREF_338)) | Inconsistent with the theme |
| Nuño T | 2011 | ([339](#_ENREF_339)) | Inconsistent with the theme |
| Lowery JT | 2011 | ([340](#_ENREF_340)) | Inconsistent with the theme |
| Lairson DR | 2011 | ([341](#_ENREF_341)) | Inconsistent with the theme |
| Hoff SR | 2011 | ([342](#_ENREF_342)) | Inconsistent with the theme |
| Dinkelspiel H | 2011 | ([343](#_ENREF_343)) | Inconsistent with the theme |
| Corsetti V | 2011 | ([344](#_ENREF_344)) | Inconsistent with the theme |
| Chlebowski R | 2011 | ([345](#_ENREF_345)) | Letter |
| Cardarelli K | 2011 | ([346](#_ENREF_346)) | Inconsistent with the theme |
| Bennett RL | 2011 | ([347](#_ENREF_347)) | Inconsistent with the theme |
| Bennett ML | 2011 | ([348](#_ENREF_348)) | Inconsistent with the theme |
| Banik S | 2011 | ([349](#_ENREF_349)) | Inconsistent with the theme  (dissertation thesis) |
| Wu JC-Y | 2010 | ([350](#_ENREF_350)) | Inconsistent with the theme |
| Woolcott CG | 2010 | ([351](#_ENREF_351)) | Inconsistent with the theme |
| Törnberg S | 2010 | ([352](#_ENREF_352)) | Inconsistent with the theme |
| Russell KM | 2010 | ([353](#_ENREF_353)) | Inconsistent with the theme |
| Ravaioli A | 2010 | ([354](#_ENREF_354)) | Inconsistent with the theme |
| Rangayyan RM | 2010 | ([355](#_ENREF_355)) | Inconsistent with the theme |
| Kuhl C | 2010 | ([356](#_ENREF_356)) | Inconsistent with the theme |
| Kingston N | 2010 | ([357](#_ENREF_357)) | Inconsistent with the theme |
| Domingo L | 2010 | ([358](#_ENREF_358)) | Inconsistent with the theme |
| Huang C | 2010 | ([118](#_ENREF_118)) | Inconsistent with the theme |
| Chlebowski RT | 2010 | ([359](#_ENREF_359)) | Inconsistent with the theme |
| Chiu SY | 2010 | ([360](#_ENREF_360)) | Inconsistent with the theme |
| Caumo F | 2010 | ([361](#_ENREF_361)) | Inconsistent with the theme |
| Vujovic O | 2009 | ([362](#_ENREF_362)) | Inconsistent with the theme |
| Stewart SL | 2009 | ([363](#_ENREF_363)) | Inconsistent with the theme |
| Sperati A, | 2009 | ([364](#_ENREF_364)) | Inconsistent with the theme |
| Seigneurin A | 2009 | ([365](#_ENREF_365)) | Inconsistent with the theme |
| Rejnmark L | 2009 | ([366](#_ENREF_366)) | Inconsistent with the theme |
| Park CC | 2009 | ([367](#_ENREF_367)) | Inconsistent with the theme |
| Nelson HD | 2009 | ([368](#_ENREF_368)) | Inconsistent with the theme |
| Malich A | 2009 | ([369](#_ENREF_369)) | Inconsistent with the theme |
| Hofvind S | 2009 | ([370](#_ENREF_370)) | Inconsistent with the theme |
| Crandall CJ | 2009 | ([371](#_ENREF_371)) | Inconsistent with the theme |
| Caumo F | 2009 | ([372](#_ENREF_372)) | Inconsistent with the theme |
| Bordas P | 2009 | ([373](#_ENREF_373)) | Inconsistent with the theme |
| Beaver K | 2009 | ([374](#_ENREF_374)) | Inconsistent with the theme |
| Tchou J | 2008 | ([375](#_ENREF_375)) | Inconsistent with the theme |
| Suzuki A | 2008 | ([376](#_ENREF_376)) | Inconsistent with the theme |
| Shen Y | 2008 | ([377](#_ENREF_377)) | Inconsistent with the theme |
| Lin K | 2008 | ([378](#_ENREF_378)) | Inconsistent with the theme |
| Kellen E | 2008 | ([379](#_ENREF_379)) | Inconsistent with the theme |
| Jandorf L | 2008 | ([380](#_ENREF_380)) | Inconsistent with the theme |
| Hofvind S | 2008 | ([381](#_ENREF_381)) | Inconsistent with the theme |
| Daguet E | 2008 | ([382](#_ENREF_382)) | Inconsistent with the theme |
| Ciatto S | 2008 | ([383](#_ENREF_383)) | Inconsistent with the theme |
| Bucchi L | 2008 | ([384](#_ENREF_384)) | Inconsistent with the theme |
| Bucchi L | 2008 | ([385](#_ENREF_385)) | Inconsistent with the theme |
| Berg WA | 2008 | ([386](#_ENREF_386)) | Inconsistent with the theme |
| Skaane P | 2007 | ([387](#_ENREF_387)) | Inconsistent with the theme |
| Porter GJR | 2007 | ([388](#_ENREF_388)) | Inconsistent with the theme |
| Porter GJR | 2007 | ([389](#_ENREF_389)) | Inconsistent with the theme |
| Mishra SI | 2007 | ([390](#_ENREF_390)) | Inconsistent with the theme |
| Han PK | 2007 | ([391](#_ENREF_391)) | Inconsistent with the theme |
| Hagen AI | 2007 | ([392](#_ENREF_392)) | Inconsistent with the theme |
| Goel A | 2007 | ([393](#_ENREF_393)) | Inconsistent with the theme |
| Evans AJ | 2007 | ([394](#_ENREF_394)) | Inconsistent with the theme |
| Dietrich AJ | 2007 | ([395](#_ENREF_395)) | Inconsistent with the theme |
| Ciatto S | 2007 | ([396](#_ENREF_396)) | Inconsistent with the theme |
| Chamot E | 2007 | ([397](#_ENREF_397)) | Inconsistent with the theme |
| Bordas P | 2007 | ([398](#_ENREF_398)) | Inconsistent with the theme |
| Zackrisson S | 2006 | ([399](#_ENREF_399)) | Inconsistent with the theme |
| Zackrisson S | 2006 | ([400](#_ENREF_400)) | Inconsistent with the theme  (dissertation thesis) |
| Vettorazzi M | 2006 | ([401](#_ENREF_401)) | Inconsistent with the theme |
| Stefanick ML | 2006 | ([402](#_ENREF_402)) | Inconsistent with the theme |
| Porter GJ | 2006 | ([403](#_ENREF_403)) | Inconsistent with the theme |
| Paskett E | 2006 | ([404](#_ENREF_404)) | Inconsistent with the theme |
| Hofvind S | 2006 | ([405](#_ENREF_405)) | Inconsistent with the theme |
| Hofvind S | 2006 | ([406](#_ENREF_406)) | Inconsistent with the theme |
| Emdin SO | 2006 | ([407](#_ENREF_407)) | Inconsistent with the theme |
| Bulliard J-L | 2006 | ([408](#_ENREF_408)) | Inconsistent with the theme |
| Anonymous | 2006 | ([409](#_ENREF_409)) | Inconsistent with the theme |
| Törnberg S | 2005 | ([410](#_ENREF_410)) | Inconsistent with the theme |
| Shen Y | 2005 | ([411](#_ENREF_411)) | Inconsistent with the theme |
| Moss S | 2005 | ([412](#_ENREF_412)) | Inconsistent with the theme |
| McTiernan A | 2005 | ([413](#_ENREF_413)) | Inconsistent with the theme |
| McAlearney AS | 2005 | ([414](#_ENREF_414)) | Inconsistent with the theme |
| Lowery JT | 2005 | ([415](#_ENREF_415)) | Inconsistent with the theme  (dissertation thesis) |
| Kerlikowske K | 2005 | ([416](#_ENREF_416)) | Inconsistent with the theme |
| Collett K | 2005 | ([417](#_ENREF_417)) | Inconsistent with the theme |
| Carney PA | 2005 | ([418](#_ENREF_418)) | Inconsistent with the theme |
| Burani R | 2005 | ([419](#_ENREF_419)) | Inconsistent with the theme |
| Abood DA | 2005 | ([420](#_ENREF_420)) | Inconsistent with the theme |
| Taylor R | 2004 | ([421](#_ENREF_421)) | Inconsistent with the theme |
| Collins LC | 2004 | ([422](#_ENREF_422)) | Inconsistent with the theme |
| Ciatto S | 2004 | ([423](#_ENREF_423)) | Inconsistent with the theme |
| Barton MB | 2004 | ([424](#_ENREF_424)) | Inconsistent with the theme |
| Anderson TJ | 2004 | ([425](#_ENREF_425)) | Inconsistent with the theme |
| Vogt TM, | 2003 | ([426](#_ENREF_426)) | Inconsistent with the theme |
| Lee SJ | 2003 | ([427](#_ENREF_427)) | Inconsistent with the theme |
| Ciatto S | 2003 | ([428](#_ENREF_428)) | Inconsistent with the theme |
| Bjurstam N | 2003 | ([429](#_ENREF_429)) | Inconsistent with the theme |
| Anttinen J | 2003 | ([430](#_ENREF_430)) | Inconsistent with the theme |
| 김준영 | 2002 | ([431](#_ENREF_431)) | Inconsistent with the theme |
| Zappa M | 2002 | ([432](#_ENREF_432)) | Inconsistent with the theme |
| Thomas DB | 2002 | ([433](#_ENREF_433)) | Inconsistent with the theme |
| Terry PD | 2002 | ([434](#_ENREF_434)) | Inconsistent with the theme |
| Taylor R | 2002 | ([435](#_ENREF_435)) | Inconsistent with the theme |
| Taplin SH | 2002 | ([436](#_ENREF_436)) | Inconsistent with the theme |
| Stoddard AM | 2002 | ([437](#_ENREF_437)) | Inconsistent with the theme |
| Reuben DB | 2002 | ([438](#_ENREF_438)) | Inconsistent with the theme |
| Gower-Thomas K | 2002 | ([439](#_ENREF_439)) | Inconsistent with the theme |
| Gao F | 2002 | ([440](#_ENREF_440)) | Inconsistent with the theme |
| Crane CEB | 2002 | ([441](#_ENREF_441)) | Inconsistent with the theme |
| Champion VL | 2002 | ([442](#_ENREF_442)) | Inconsistent with the theme |
| Wang H | 2001 | ([443](#_ENREF_443)) | Inconsistent with the theme |
| Raja MA | 2001 | ([444](#_ENREF_444)) | Inconsistent with the theme |
| Moberg K | 2001 | ([445](#_ENREF_445)) | Inconsistent with the theme |
| McCann J | 2001 | ([446](#_ENREF_446)) | Inconsistent with the theme |
| Kaas R | 2001 | ([447](#_ENREF_447)) | Inconsistent with the theme |
| Garvican L | 2001 | ([448](#_ENREF_448)) | Inconsistent with the theme |
| Ganry OF | 2001 | ([449](#_ENREF_449)) | Inconsistent with the theme |
| Ellis PM | 2001 | ([450](#_ENREF_450)) | Inconsistent with the theme |
| Brown M | 2001 | ([451](#_ENREF_451)) | Inconsistent with the theme |
| Britton PD | 2001 | ([452](#_ENREF_452)) | Inconsistent with the theme |
| Allen JD | 2001 | ([453](#_ENREF_453)) | Inconsistent with the theme |
| Banks E | 2001 | ([95](#_ENREF_95)) | Review |
| Warren R | 2000 | ([454](#_ENREF_454)) | Inconsistent with the theme |
| Taplin SH | 2000 | ([455](#_ENREF_455)) | Inconsistent with the theme |
| Seger S | 2000 | ([456](#_ENREF_456)) | Inconsistent with the theme |
| Morrone D | 2000 | ([457](#_ENREF_457)) | Inconsistent with the theme |
| Miller AB | 2000 | ([458](#_ENREF_458)) | Inconsistent with the theme |
| Mandelson MT | 2000 | ([459](#_ENREF_459)) | Inconsistent with the theme |
| Gilliland FD | 2000 | ([460](#_ENREF_460)) | Inconsistent with the theme |
| Eisinger F | 2000 | ([461](#_ENREF_461)) | Letter |
| de Rijke JM | 2000 | ([462](#_ENREF_462)) | Inconsistent with the theme |
| Crane LA | 2000 | ([463](#_ENREF_463)) | Inconsistent with the theme |
| Cowan WK | 2000 | ([464](#_ENREF_464)) | Inconsistent with the theme |
| Amos AF | 2000 | ([465](#_ENREF_465)) | Inconsistent with the theme |
| Vitak B | 1999 | ([466](#_ENREF_466)) | Inconsistent with the theme |
| Taylor V | 1999 | ([467](#_ENREF_467)) | Inconsistent with the theme |
| Tabar L | 1999 | ([468](#_ENREF_468)) | Inconsistent with the theme |
| Porter PL | 1999 | ([469](#_ENREF_469)) | Inconsistent with the theme |
| Moberg K | 1999 | ([470](#_ENREF_470)) | Inconsistent with the theme |
| Michaelson JS | 1999 | ([471](#_ENREF_471)) | Inconsistent with the theme |
| Liljegren G | 1999 | ([472](#_ENREF_472)) | Inconsistent with the theme |
| Kavanagh AM | 1999 | ([473](#_ENREF_473)) | Inconsistent with the theme |
| Exbrayat C | 1999 | ([474](#_ENREF_474)) | Inconsistent with the theme |
| Burman ML | 1999 | ([475](#_ENREF_475)) | Inconsistent with the theme |
| Vitak B | 1998 | ([476](#_ENREF_476)) | Inconsistent with the theme |
| Thurfjell E | 1998 | ([477](#_ENREF_477)) | Inconsistent with the theme |
| Rickard MT | 1998 | ([478](#_ENREF_478)) | Inconsistent with the theme |
| Ng EH | 1998 | ([479](#_ENREF_479)) | Inconsistent with the theme |
| Moss S | 1998 | ([480](#_ENREF_480)) | Inconsistent with the theme |
| de Korvin B | 1998 | ([481](#_ENREF_481)) | Inconsistent with the theme |
| Bird JA | 1998 | ([482](#_ENREF_482)) | Inconsistent with the theme |
| Weber BE | 1997 | ([483](#_ENREF_483)) | Inconsistent with the theme |
| Vitak B | 1997 | ([484](#_ENREF_484)) | Inconsistent with the theme |
| Sylvester PA | 1997 | ([485](#_ENREF_485)) | Inconsistent with the theme |
| Sylvester PA | 1997 | ([486](#_ENREF_486)) | Inconsistent with the theme |
| Paci E | 1997 | ([487](#_ENREF_487)) | Inconsistent with the theme |
| Miller AB | 1997 | ([488](#_ENREF_488)) | Inconsistent with the theme |
| Lenner P | 1997 | ([489](#_ENREF_489)) | Inconsistent with the theme |
| Larsson LG | 1997 | ([490](#_ENREF_490)) | Inconsistent with the theme |
| Klemi PJ | 1997 | ([491](#_ENREF_491)) | Inconsistent with the theme |
| Hendrick RE | 1997 | ([492](#_ENREF_492)) | Inconsistent with the theme |
| Hakama M | 1997 | ([493](#_ENREF_493)) | Inconsistent with the theme |
| Frisell J | 1997 | ([494](#_ENREF_494)) | Inconsistent with the theme |
| Frisell J | 1997 | ([495](#_ENREF_495)) | Inconsistent with the theme |
| Boyd NF | 1997 | ([496](#_ENREF_496)) | Inconsistent with the theme |
| Bjurstam N | 1997 | ([497](#_ENREF_497)) | Inconsistent with the theme |
| Vizcaino Esteve I | 1996 | ([498](#_ENREF_498)) | Inconsistent with the theme |
| Burrell HC | 1996 | ([499](#_ENREF_499)) | Inconsistent with the theme |
| Beaulieu MD | 1996 | ([500](#_ENREF_500)) | Inconsistent with the theme |
| Asbury D | 1996 | ([501](#_ENREF_501)) | Inconsistent with the theme |
| Woodman CB | 1995 | ([502](#_ENREF_502)) | Inconsistent with the theme |
| Wald NJ | 1995 | ([503](#_ENREF_503)) | Inconsistent with the theme |
| Schaffer P | 1995 | ([504](#_ENREF_504)) | Inconsistent with the theme |
| Kopans DB | 1995 | ([505](#_ENREF_505)) | Letter |
| Kerlikowske K | 1995 | ([506](#_ENREF_506)) | Inconsistent with the theme |
| Goff JM | 1995 | ([507](#_ENREF_507)) | Inconsistent with the theme |
| Field S | 1995 | ([508](#_ENREF_508)) | Inconsistent with the theme |
| Duncan AA | 1995 | ([509](#_ENREF_509)) | Inconsistent with the theme |
| Day N | 1995 | ([510](#_ENREF_510)) | Inconsistent with the theme |
| Brekelmans CT | 1995 | ([511](#_ENREF_511)) | Inconsistent with the theme |
| Boer R | 1995 | ([512](#_ENREF_512)) | Letter |
| Spiegelman D | 1994 | ([513](#_ENREF_513)) | Inconsistent with the theme |
| Meldrum P | 1994 | ([514](#_ENREF_514)) | Inconsistent with the theme |
| Liljegren G | 1994 | ([515](#_ENREF_515)) | Inconsistent with the theme |
| Koivunen D | 1994 | ([516](#_ENREF_516)) | Inconsistent with the theme |
| Kiefe CI | 1994 | ([517](#_ENREF_517)) | Inconsistent with the theme |
| Frischbier HJ | 1994 | ([518](#_ENREF_518)) | Inconsistent with the theme |
| Burhenne HJ | 1994 | ([519](#_ENREF_519)) | Inconsistent with the theme |
| Brekelmans CT | 1994 | ([520](#_ENREF_520)) | Inconsistent with the theme |
| Baines CJ | 1994 | ([521](#_ENREF_521)) | Inconsistent with the theme |
| Arnesson LG | 1994 | ([522](#_ENREF_522)) | Inconsistent with the theme |
| Watmough DJ | 1993 | ([523](#_ENREF_523)) | Comment |
| Vandenbroucke A | 1993 | ([524](#_ENREF_524)) | Inconsistent with the theme |
| van Dijck JA | 1993 | ([525](#_ENREF_525)) | Inconsistent with the theme |
| Tabar L | 1993 | ([526](#_ENREF_526)) | Inconsistent with the theme |
| Nyström L | 1993 | ([527](#_ENREF_527)) | Inconsistent with the theme |
| Moss SM | 1993 | ([528](#_ENREF_528)) | Inconsistent with the theme |
| Holowaty PH | 1993 | ([529](#_ENREF_529)) | Inconsistent with the theme |
| Fletcher SW | 1993 | ([530](#_ENREF_530)) | Inconsistent with the theme |
| Miller AB | 1992 | ([531](#_ENREF_531)) | Inconsistent with the theme |
| Frisell J | 1992 | ([532](#_ENREF_532)) | Inconsistent with the theme |
| Frisell J | 1911 | ([533](#_ENREF_533)) | Inconsistent with the theme |
| Cuckle H | 1911 | ([534](#_ENREF_534)) | Inconsistent with the theme |
| Peeters PH | 1990 | ([535](#_ENREF_535)) | Inconsistent with the theme |
| Baines CJ | 1990 | ([536](#_ENREF_536)) | Inconsistent with the theme |
| Peeters PH | 1989 | ([537](#_ENREF_537)) | Inconsistent with the theme |
| Lewars MD | 2002 | ([538](#_ENREF_538)) | Letter |
| Narod SA | 2001 | ([539](#_ENREF_539)) | Letter |
| Pulido-Carmona C | 2024 | ([540](#_ENREF_540)) | Meeting the Inclusion Criteria |
| Pattacini P | 2022 | ([541](#_ENREF_541)) | Meeting the Inclusion Criteria |
| Armaroli P | 2022 | ([542](#_ENREF_542)) | Meeting the Inclusion Criteria |
| Hofvind S | 2021 | ([543](#_ENREF_543)) | Meeting the Inclusion Criteria |
| Winter AM | 2020 | ([544](#_ENREF_544)) | Meeting the Inclusion Criteria |
| Hovda T | 2020 | ([545](#_ENREF_545)) | Meeting the Inclusion Criteria |
| Bernardi D | 2020 | ([546](#_ENREF_546)) | Meeting the Inclusion Criteria |
| Hovda T | 2019 | ([547](#_ENREF_547)) | Meeting the Inclusion Criteria |
| Skaane P | 2018 | ([548](#_ENREF_548)) | Meeting the Inclusion Criteria |
| Houssami N | 2018 | ([549](#_ENREF_549)) | Meeting the Inclusion Criteria |
| McDonald ES | 2016 | ([550](#_ENREF_550)) | Meeting the Inclusion Criteria |

**References：**

1. Moshina N, Grawingholt A, Lang K, Mann R, Hovda T, Hoff SR, et al. Digital breast tomosynthesis in mammographic screening: false negative cancer cases in the To-Be 1 trial. Insights into imaging. 2024;15(1).

2. Giorgi Rossi P, Mancuso P, Pattacini P, Campari C, Nitrosi A, Iotti V, et al. Comparing accuracy of tomosynthesis plus digital mammography or synthetic 2D mammography in breast cancer screening: baseline results of the MAITA RCT consortium. Eur J Cancer. 2024;199:113553.

3. Majid SZ, Senapati GM, Lacson R, Chikarmane SA, Giess CS. Imaging characteristics of interval cancers detected on Full Field Digital Mammography (FFDM) versus Digital Breast Tomosynthesis (DBT). Clinical Imaging. 2024;107.

4. Orsini L, Czene K, Humphreys K. Random effects models of tumour growth for investigating interval breast cancer. Stat Med. 2024;43(15):2957-71.

5. Nykanen A, Sudah M, Masarwah A, Vanninen R, Okuma H. Radiological features of screening-detected and interval breast cancers and subsequent survival in Eastern Finnish women. Scientific Reports. 2024;14(1).

6. Mullen LA. Can digital breast tomosynthesis decrease interval cancers in a breast cancer screening program? European Radiology. 2024.

7. Celik L, Aribal E. The efficacy of artificial intelligence (AI) in detecting interval cancers in the national screening program of a middle-income country. Clinical Radiology. 2024;79(7):e885-e91.

8. ten Velde DE, Duijm LEM, van der Sangen MJC, Schipper R-J, Tjan-Heijnen VCG, Vreuls W, et al. Long-term trends in incidence, characteristics and prognosis of screen-detected and interval cancers in women participating in the Dutch breast cancer screening programme. British Journal of Cancer. 2024;130(9):1561-70.

9. Song H, Tran TXM, Kim S, Park B. Risk Factors and Mortality Among Women With Interval Breast Cancer vs Screen-Detected Breast Cancer. JAMA Netw Open. 2024;7(5):e2411927.

10. Sprague BL, Coley RY, Lowry KP, Kerlikowske K, Henderson LM, Su YR, et al. Digital Breast Tomosynthesis versus Digital Mammography Screening Performance on Successive Screening Rounds from the Breast Cancer Surveillance Consortium. Radiology. 2023;307(5):e223142.

11. Skaane P. Interval and Successive-Round Cancers after Digital Breast Tomosynthesis Screening: We Still Need Convincing Results regarding Beneficial Evidence on Long-term Outcomes. Radiology. 2023;307(5).

12. Shima A, Tanaka H, Okamura T, Nishikawa T, Morino A, Godai K, et al. Offering on-site mammography in workplaces improved screening rates: cluster randomized controlled trial. Journal of occupational health. 2023;65(1):e12389.

13. Ramadas K, Basu P, Mathew BS, Muwonge R, Venugopal M, Prakasan AM, et al. Effectiveness of triennial screening with clinical breast examination: 14-years follow-up outcomes of randomized clinical trial in Trivandrum, India. Cancer. 2023;129(2):272‐82.

14. Larsen M, Lynge E, Lee CI, Lang K, Hofvind S. Mammographic density and interval cancers in mammographic screening: Moving towards more personalized screening. Breast. 2023;69:306-11.

15. Lång K, Josefsson V, Larsson AM, Larsson S, Högberg C, Sartor H, et al. Artificial intelligence-supported screen reading versus standard double reading in the Mammography Screening with Artificial Intelligence trial (MASAI): a clinical safety analysis of a randomised, controlled, non-inferiority, single-blinded, screening accuracy study. The lancet Oncology. 2023;24(8):936‐44.

16. Vachon CM, Scott CG, Norman AD, Khanani SA, Jensen MR, Hruska CB, et al. Impact of Artificial Intelligence System and Volumetric Density on Risk Prediction of Interval, Screen-Detected, and Advanced Breast Cancer. Journal of Clinical Oncology. 2023;41(17):3172-+.

17. Holen Å, Bergan MB, Lee CI, Zackrisson S, Moshina N, Aase HS, et al. Early screening outcomes before, during, and after a randomized controlled trial with digital breast tomosynthesis. European journal of radiology. 2023;167:111069.

18. Heinze F, Czwikla J, Heinig M, Langner I, Haug U. German mammography screening program: program sensitivity between 2010 and 2016 estimated based on German health claims data. BMC Cancer. 2023;23(1):852.

19. Moger TA, Holen A, Hanestad B, Hofvind S. Costs and Effects of Implementing Digital Tomosynthesis in a Population-Based Breast Cancer Screening Program: predictions Using Results from the To-Be Trial in Norway. PharmacoEconomics - open. 2022;6(4):495‐507.

20. Kerlikowske K, Su Y-R, Sprague BL, Tosteson ANA, Buist DSM, Onega T, et al. Association of Screening With Digital Breast Tomosynthesis vs Digital Mammography With Risk of Interval Invasive and Advanced Breast Cancer. Jama-Journal of the American Medical Association. 2022;327(22):2220-30.

21. Heindel W, Weigel S, Kerschke L, Baier S, Sommer A, Czwoydzinski J, et al. Breast cancer screening: tomosynthesis plus synthesised mammograms versus digital mammography: first results of the TOSYMA RCT. Insights into imaging. 2022;14:423.

22. Heindel W, Weigel S, Gerß J, Hense HW, Sommer A, Krischke M, et al. Digital breast tomosynthesis plus synthesised mammography versus digital screening mammography for the detection of invasive breast cancer (TOSYMA): a multicentre, open-label, randomised, controlled, superiority trial. The lancet Oncology. 2022;23(5):601‐11.

23. Zhang Z, Tabung FK, Jin Q, Curran G, Irvin VL, Shannon J, et al. Diet-Driven Inflammation and Insulinemia and Risk of Interval Breast Cancer. Nutrition and Cancer-an International Journal. 2022;74(9):3179-93.

24. Zhang Z, Curran G, Shannon J, Velie EM, Irvin VL, Manson JE, et al. Body Mass Index Is Inversely Associated with Risk of Postmenopausal Interval Breast Cancer: Results from the Women's Health Initiative. Cancers (Basel). 2022;14(13).

25. Wanders AJT, Mees W, Bun PAM, Janssen N, Rodriguez-Ruiz A, Dalmis MU, et al. Interval Cancer Detection Using a Neural Network and Breast Density in Women with Negative Screening Mammograms. Radiology. 2022;303(2).

26. Ugalde-Morales E, Grassmann F, Humphreys K, Li J, Eriksson M, Tobin NP, et al. Interval breast cancer is associated with interferon immune response. Eur J Cancer. 2022;162:194-205.

27. Timmermans L, De Brabander I, Van Damme N, Bleyen L, Martens P, Van Herck K, et al. Tumour characteristics of screen-detected and interval cancers in the Flemish Breast Cancer Screening Programme: A mammographic breast density study. Maturitas. 2022;158:55-60.

28. Seely JM, Peddle SE, Yang H, Chiarelli AM, McCallum M, Narasimhan G, et al. Breast Density and Risk of Interval Cancers: The Effect of Annual Versus Biennial Screening Mammography Policies in Canada. Canadian Association of Radiologists Journal-Journal De L Association Canadienne Des Radiologistes. 2022;73(1):90-100.

29. Ryser MD, Lange J, Inoue LYT, O'Meara ES, Gard C, Miglioretti DL, et al. Estimation of Breast Cancer Overdiagnosis in a U.S. Breast Screening Cohort. Ann Intern Med. 2022;175(4):471-8.

30. Noguchi N, Marinovich ML, Wylie EJ, Lund HG, Houssami N. Evidence from a BreastScreen cohort does not support a longer inter-screen interval in women who have no conventional risk factors for breast cancer. Breast. 2022;62:16-21.

31. Hovda T, Hoff SR, Larsen M, Romundstad L, Sahlberg KK, Hofvind S. True and Missed Interval Cancer in Organized Mammographic Screening: A Retrospective Review Study of Diagnostic and Prior Screening Mammograms. Acad Radiol. 2022;29 Suppl 1:S180-s91.

32. Friedewald SM, Grimm LJ. Digital Breast Tomosynthesis and Detection of Interval Invasive and Advanced Breast Cancers. Jama-Journal of the American Medical Association. 2022;327(22):2198-200.

33. Byng D, Strauch B, Gnas L, Leibig C, Stephan O, Bunk S, et al. AI-based prevention of interval cancers in a national mammography screening program. Eur J Radiol. 2022;152:110321.

34. Martiniussen MA, Sagstad S, Larsen M, Larsen ASF, Hovda T, Lee CI, et al. Screen-detected and interval breast cancer after concordant and discordant interpretations in a population based screening program using independent double reading. Eur Radiol. 2022;32(9):5974-85.

35. Johnson K, Lång K, Ikeda DM, Åkesson A, Andersson I, Zackrisson S. Interval Breast Cancer Rates and Tumor Characteristics in the Prospective Population-based Malmö Breast Tomosynthesis Screening Trial. Radiology. 2021;299(3):559-67.

36. Zhu X, Wolfgruber TK, Leong L, Jensen M, Scott C, Winham S, et al. Deep Learning Predicts Interval and Screening-detected Cancer from Screening Mammograms: A Case-Case-Control Study in 6369 Women. Radiology. 2021;301(3):550-8.

37. Taourel P. Interval Breast Cancer after Digital Breast Tomosynthesis-based Screening: A Glimmer of Hope. Radiology. 2021;300(1):77-8.

38. Nguyen TL, Schmidt DF, Makalic E, Maskarinec G, Li S, Dite GS, et al. Novel mammogram-based measures improve breast cancer risk prediction beyond an established mammographic density measure. Int J Cancer. 2021;148(9):2193-202.

39. Lang K, Hofvind S, Rodriguez-Ruiz A, Andersson I. Can artificial intelligence reduce the interval cancer rate in mammography screening? European Radiology. 2021;31(8):5940-7.

40. Harada-Shoji N, Suzuki A, Ishida T, Zheng YF, Narikawa-Shiono Y, Sato-Tadano A, et al. Evaluation of Adjunctive Ultrasonography for Breast Cancer Detection among Women Aged 40-49 Years with Varying Breast Density Undergoing Screening Mammography: a Secondary Analysis of a Randomized Clinical Trial. JAMA network open. 2021;4(8):e2121505.

41. Graewingholt A, Rossi PG. Retrospective analysis of the effect on interval cancer rate of adding an artificial intelligence algorithm to the reading process for two-dimensional full-field digital mammography. Journal of Medical Screening. 2021;28(3):369-71.

42. van Zelst JC, Tan T, Mann RM, Karssemeijer N. Validation of radiologists' findings by computer-aided detection (CAD) software in breast cancer detection with automated 3D breast ultrasound: a concept study in implementation of artificial intelligence software. Acta radiologica (Stockholm, Sweden : 1987). 2020;61(3):312‐20.

43. Shieh Y, Ziv E, Kerlikowske K. Interval breast cancers - insights into a complex phenotype. Nature Reviews Clinical Oncology. 2020;17(3):138-9.

44. Pisano E, Gatsonis C, Yaffe M, Troester M, Gareen IF, Collins LC, et al. ECOG-ACRIN tomosynthesis mammographic imaging screening trial (EA1151). Journal of clinical oncology. 2020;38(15).

45. Njor SH, Vejborg I, Larsen MB. Breast cancer survivors' risk of interval cancers and false positive results in organized mammography screening. Cancer Medicine. 2020;9(16):6042-50.

46. Niraula S, Biswanger N, Hu P, Lambert P, Decker K. Incidence, Characteristics, and Outcomes of Interval Breast Cancers Compared With Screening-Detected Breast Cancers. JAMA Netw Open. 2020;3(9):e2018179.

47. Nguyen TL, Li S, Dite GS, Aung YK, Evans CF, Trinh HN, et al. Interval breast cancer risk associations with breast density, family history and breast tissue aging. International Journal of Cancer. 2020;147(2):375-82.

48. Luker GD. MRI Screening Reduces Interval Breast Cancer in Women with Dense Breasts. Radiol Imaging Cancer. 2020;2(1):e204002.

49. Comstock CE, Gatsonis C, Newstead GM, Snyder BS, Gareen IF, Bergin JT, et al. Comparison of Abbreviated Breast MRI vs Digital Breast Tomosynthesis for Breast Cancer Detection Among Women With Dense Breasts Undergoing Screening. JAMA. 2020;323(8):746‐56.

50. Román M, Hofvind S, von Euler-Chelpin M, Castells X. Long-term risk of screen-detected and interval breast cancer after false-positive results at mammography screening: joint analysis of three national cohorts. Br J Cancer. 2019;120(2):269-75.

51. Pilewskie M, Zabor EC, Gilbert E, Stempel M, Petruolo O, Mangino D, et al. Differences between screen-detected and interval breast cancers among BRCA mutation carriers. Breast Cancer Research and Treatment. 2019;175(1):141-8.

52. Hofvind S, Holen Å, Aase HS, Houssami N, Sebuødegård S, Moger TA, et al. Two-view digital breast tomosynthesis versus digital mammography in a population-based breast cancer screening programme (To-Be): a randomised, controlled trial. The lancet Oncology. 2019;20(6):795‐805.

53. Tilanus-Linthorst MM, Saadatmand S, Geuzinge AH, Rutgers EJ, Mann R, De Roy Van Zuidewijn DB, et al. MRI breast cancer screening compared to mammography in women with a familial risk: a multicenter randomized controlled trial. Cancer research. 2019;79(4).

54. Steponaviciene L, Vincerzevskiene I, Briediene R, Urbonas V, Vanseviciute-Petkeviciene R, Smailyte G. Breast Cancer Screening Program in Lithuania: Interval Cancers and Program Sensitivity After 7 Years of Mammography Screening. Cancer Control. 2019;26(1).

55. Weigel S, Gerss J, Hense HW, Krischke M, Sommer A, Czwoydzinski J, et al. Digital breast tomosynthesis plus synthesised images versus standard full-field digital mammography in population-based screening (TOSYMA): protocol of a randomised controlled trial. BMJ open. 2018;8(5):e020475.

56. Pattacini P, Nitrosi A, Giorgi Rossi P, Iotti V, Ginocchi V, Ravaioli S, et al. Digital Mammography versus Digital Mammography Plus Tomosynthesis for Breast Cancer Screening: the Reggio Emilia Tomosynthesis Randomized Trial. Radiology. 2018;288(2):375‐85.

57. Bahl M, Gaffney S, McCarthy AM, Lowry KP, Dang PA, Lehman CD. Breast Cancer Characteristics Associated with 2D Digital Mammography versus Digital Breast Tomosynthesis for Screening-detected and Interval Cancers. Radiology. 2018;287(1):49-57.

58. Hofvind S, Sagstad S, Sebuødegård S, Chen Y, Roman M, Lee CI. Interval Breast Cancer Rates and Histopathologic Tumor Characteristics after False-Positive Findings at Mammography in a Population-based Screening Program. Radiology. 2018;287(1):58-67.

59. Sayed S, Ngugi AK, Nwosu N, Mutebi MC, Ochieng P, Mwenda AS, et al. Training health workers in clinical breast examination for early detection of breast cancer in low‐ and middle‐income countries. Cochrane Database of Systematic Reviews. 2023(4).

60. Glechner A, Wagner G, Mitus JW, Teufer B, Klerings I, Böck N, et al. Mammography in combination with breast ultrasonography versus mammography for breast cancer screening in women at average risk. Cochrane Database of Systematic Reviews. 2023(3).

61. Zeng A, Brennan ME, Young S, Mathieu E, Houssami N. The Effect of Supplemental Imaging on Interval Cancer Rates in Mammography Screening: Systematic Review. Clinical Breast Cancer. 2022;22(3):212-22.

62. Hovda T, Hoff SR, Larsen M, Romundstad L, Sahlberg KK, Hofvind S. True and Missed Interval Cancer in Organized Mammographic Screening: A Retrospective Review Study of Diagnostic and Prior Screening Mammograms. Academic Radiology. 2022;29:S180-S91.

63. Farber R, Houssami N, Barnes I, McGeechan K, Barratt A, Bell KJL. Considerations for Evaluating the Introduction of New Cancer Screening Technology: Use of Interval Cancers to Assess Potential Benefits and Harms. International Journal of Environmental Research and Public Health. 2022;19(22).

64. Tsuruda KM, Hovda T, Bhargava S, Veierod MB, Hofvind S. Survival among women diagnosed with screen-detected or interval breast cancer classified as true, minimal signs, or missed through an informed radiological review. European Radiology. 2021;31(5):2677-86.

65. Mullooly M, White G, Bennett K, O'Doherty A, Flanagan F, Healy O. Retrospective radiological review and classification of interval breast cancers within population-based breast screening programmes for the purposes of open disclosure: A systematic review. European Journal of Radiology. 2021;138.

66. Houssami N, Zackrisson S, Blazek K, Hunter K, Bernardi D, Lang K, et al. Meta-analysis of prospective studies evaluating breast cancer detection and interval cancer rates for digital breast tomosynthesis versus mammography population screening. European Journal of Cancer. 2021;148:14-23.

67. Houssami N, Hofvind S, Soerensen AL, Robledo KP, Hunter K, Bernardi D, et al. Interval breast cancer rates for digital breast tomosynthesis versus digital mammography population screening: An individual participant data meta-analysis. EClinicalMedicine. 2021;34:100804.

68. Messinger J, Crawford S, Roland L, Mizuguchi S. Review of Subtypes of Interval Breast Cancers With Discussion of Radiographic Findings. Current problems in diagnostic radiology. 2019;48(6):592-8.

69. Mandoul C, Verheyden C, Millet I, Orliac C, Pages E, Thomassin I, et al. Breast tomosynthesis: What do we know and where do we stand? Diagn Interv Imaging. 2019;100(10):537-51.

70. Destounis S, Santacroce A. Age to Begin and Intervals for Breast Cancer Screening: Balancing Benefits and Harms. American Journal of Roentgenology. 2018;210(2):279-84.

71. Lekanidi K, Dilks P, Suaris T, Kennett S, Purushothaman H. Breast screening: What can the interval cancer review teach us? Are we perhaps being a bit too hard on ourselves? European Journal of Radiology. 2017;94:13-5.

72. Houssami N, Hunter K. The epidemiology, radiology and biological characteristics of interval breast cancers in population mammography screening. Npj Breast Cancer. 2017;3.

73. Moschetti I, Cinquini M, Lambertini M, Levaggi A, Liberati A. Follow‐up strategies for women treated for early breast cancer. Cochrane Database of Systematic Reviews. 2016(5).

74. Abdel‐Aleem H, El‐Gibaly OMH, El‐Gazzar A, Al‐Attar GST. Mobile clinics for women's and children's health. Cochrane Database of Systematic Reviews. 2016(8).

75. Knox M, O'Brien A, Szabo E, Smith CS, Fenlon HM, McNicholas MM, et al. Impact of full field digital mammography on the classification and mammographic characteristics of interval breast cancers. European Journal of Radiology. 2015;84(6):1056-61.

76. Payne JI, Caines JS, Gallant J, Foley TJ. A Review of Interval Breast Cancers Diagnosed among Participants of the Nova Scotia Breast Screening Program. Radiology. 2013;266(1):96-103.

77. Gøtzsche PC, Jørgensen KJ. Screening for breast cancer with mammography. Cochrane Database of Systematic Reviews. 2013(6).

78. Goodwin A, Parker S, Ghersi D, Wilcken N. Post‐operative radiotherapy for ductal carcinoma in situ of the breast. Cochrane Database of Systematic Reviews. 2013(11).

79. Edwards AGK, Naik G, Ahmed H, Elwyn GJ, Pickles T, Hood K, et al. Personalised risk communication for informed decision making about taking screening tests. Cochrane Database of Systematic Reviews. 2013(2).

80. Hoff SR, Abrahamsen A-L, Samset JH, Vigeland E, Klepp O, Hofvind S. Breast Cancer: Missed Interval and Screening-detected Cancer at Full-Field Digital Mammography and Screen-Film Mammography-Results from a Retrospective Review. Radiology. 2012;264(2):378-86.

81. Pellegrini M, Bernardi D, Di Michele S, Tuttobene P, Fanto C, Valentini M, et al. Analysis of proportional incidence and review of interval cancer cases observed within the mammography screening programme in Trento province, Italy. Radiologia Medica. 2011;116(8):1217-25.

82. Ciatto S, Bernardi D, Caumo F. Evidence of interval cancer proportional incidence and review from mammography screening programs in Italy. Tumori. 2011;97(4):419-22.

83. Bennett ML, Welman CJ, Celliers LM. How reassuring is a normal breast ultrasound in assessment of a screen-detected mammographic abnormality? A review of interval cancers after assessment that included ultrasound evaluation. Clinical Radiology. 2011;66(10):928-39.

84. Magnus MC, Ping M, Shen MM, Bourgeois J, Magnus JH. Effectiveness of mammography screening in reducing breast cancer mortality in women aged 39-49 years: a meta-analysis. Journal of women's health (2002). 2011;20(6):845‐52.

85. Elena PM, Nehmat H, Ermes M, Piera C, Maria Q, Guia M, et al. Quality of mammography screening in the Milan programme: Evidence of improved sensitivity based on interval cancer proportional incidence and radiological review. Breast. 2009;18(3):208-10.

86. Nothacker M, Duda V, Hahn M, Warm M, Degenhardt F, Madjar H, et al. Early detection of breast cancer: benefits and risks of supplemental breast ultrasound in asymptomatic women with mammographically dense breast tissue. A systematic review. BMC Cancer. 2009;9:335.

87. Miller D, Livingstone V, Herbison GP. Interventions for relieving the pain and discomfort of screening mammography. Cochrane Database of Systematic Reviews. 2008(1).

88. Dale J, Caramlau IO, Lindenmeyer A, Williams SM. Peer support telephone calls for improving health. Cochrane Database of Systematic Reviews. 2008(4).

89. Gordon PB, Borugian MJ, Burhenne LJW. A true screening environment for review of interval breast cancers: Pilot study to reduce bias. Radiology. 2007;245(2):411-5.

90. Houssami N, Irwig L, Ciatto S. Radiological surveillance of interval breast cancers in screening programmes. Lancet Oncology. 2006;7(3):259-65.

91. Hofvind S, Skaane P, Vitak B, Wang H, Thoresen S, Eriksen L, et al. Influence of review design on percentages of missed interval breast cancers: Retrospective study of interval cancers in a population-based screening program. Radiology. 2005;237(2):437-43.

92. Baker S, Wall M, Bloomfield A. What is the most appropriate breast-cancer screening interval for women aged 45 to 49 years in New Zealand? The New Zealand medical journal. 2005;118(1221):U1636-U.

93. Ratner PA, Bottorff JL, Johnson JL, Cook R, Lovato CY. A meta-analysis of mammography screening promotion. Cancer detection and prevention. 2001;25(2):147‐60.

94. Bonfill Cosp X, Marzo Castillejo M, Pladevall Vila M, Marti J, Emparanza JI. Strategies for increasing the participation of women in community breast cancer screening. Cochrane Database of Systematic Reviews. 2001(1).

95. Banks E. Hormone replacement therapy and the sensitivity and specificity of breast cancer screening: a review. J Med Screen. 2001;8(1):29-34.

96. de Rijke JM, Schouten LJ, Schreutelkamp JL, Jochem I, Verbeek AL. A blind review and an informed review of interval breast cancer cases in the Limburg screening programme, the Netherlands. Journal of medical screening. 2000;7(1):19-23.

97. Tosteson TD, Pogue BW, Demidenko E, McBride TO, Paulsen KD. Confidence maps and confidence intervals for near infrared images in breast cancer. IEEE transactions on medical imaging. 1999;18(12):1188-93.

98. Koivunen D, Zhang X, Blackwell C, Adelstein E, Humphrey L. Interval breast cancers are not biologically distinct--just more difficult to diagnose. American journal of surgery. 1994;168(6):538-42.

99. Ray KM. Interval Cancers in Understanding Screening Outcomes. Radiologic Clinics of North America. 2024;62(4):559-69.

100. Andersen SB, Tornberg S, Lynge E, Von Euler-Chelpin M, Njor SH. A simple way to measure the burden of interval cancers in breast cancer screening. Bmc Cancer. 2014;14.

101. Summaries for Patients. Identifying women with dense breasts at high risk for interval cancer. Annals of internal medicine. 2015;162(10).

102. Acosta-Benito MA, Vich-Perez P. Interval cancer, screening, and risk markers in breast tumours. Semergen. 2016;42(8):e154-e6.

103. Liston JC. Case report: an unusual interval breast cancer masquerading as a simple cyst. Clin Radiol. 1997;52(11):876-8.

104. Sakurai K, Fujisaki S, Adachi K, Suzuki S, Masuo Y, Nagashima S, et al. Interval Breast Cancer with Neuroendocrine Differentiation. Gan to kagaku ryoho Cancer & chemotherapy. 2015;42(12):1770-2.

105. Kaplan HG, Malmgren JA, Atwood MK. Differential distant disease-free intervals for mammography detected vs. clinical presentation invasive breast cancer: Early detection or lead time bias? Cancer Research. 2019;79(4).

106. Singh D, Miettinen J, Duffy S, Malila N, Pitkaniemi J, Anttila A. Breast symptoms and risk of interval breast cancers in mammography-screening programme. European Journal of Cancer. 2018;92:S11-S.

107. Ahn S, Wooster M, Valente C, Pisapati K, Couri R, Ru M, et al. Impact of screening mammography interval on stage and treatment in women diagnosed with breast cancer. Annals of Surgical Oncology. 2018;25:281-2.

108. Czene K, Ivansson E, Klevebring D, Tobin NP, Lindstrom LS, Holm J, et al. Molecular differences between screen-detected and interval breast cancers are largely explained by PAM50 subtypes. Cancer Research. 2017;77.

109. Ferguson J, Stevens G, Hills R, Thomas KG. A comparison of interval breast cancers before and after the introduction of digital screening mammography. Breast Cancer Research. 2016;18.

110. De Munck L, De bock GH, Otter R, Reiding D, Broeders MJM, Willemse PHB, et al. Digital versus screen-film mammography in population-based breast cancer screening: Performance indicators and tumor characteristics of screen-detected and interval cancers. Cancer Research. 2016;76.

111. Jeitler K, Semlitsch T, Posch N, Siebenhofer A, Horvath K. Breast cancer screening in Austria: Key figures, age limits, screening intervals and evidence. Zeitschrift fur Evidenz, Fortbildung und Qualitat im Gesundheitswesen. 2015;109(4-5):363-70.

112. Klingen TA, Stefansson I, Collett K, Aas T, Abrahamsen AL, Aas H, et al. Interval detected breast cancers are associated with tumor cells invading blood vessels. Virchows Archiv. 2013;463(2):267-.

113. Andersen SB, Euler-Chelpin MV, Lynge E, Toernberg S, Njor SH. Can the interval cancer ratio diminish the need for background incidence estimates as a mammography screening programme performance indicator? Cancer Research. 2013;73.

114. Sala M, Domingo L, Blanch J, Bare M, Ferrer J, Fernandez AB, et al. DESCRIPTION OF A NATIONWIDE AND MULTIDISCIPLINARY PROJECT FOR THE EVALUATION OF INTERVAL BREAST CANCER RATES, DETERMINANTS AND CHARACTERISTICS, IN SPAIN. INCA STUDY. Journal of Epidemiology and Community Health. 2011;65:A103-A.

115. Musolino A, Michiara M, Conti GM, Boggiani D, Bozzani F, Zatelli M, et al. HER2 Status as Predictor of Mammographic Screening Detection: Comparison of Interval-and Screen-Detected Breast Cancers. Cancer Research. 2010;70.

116. Rayson D, Payne JI, Barnes PJ, MacIntosh RF, Abdolell M, Foley T, et al. Interval Breast Cancers Are Associated with More Aggressive Pathologic Characteristics Compared to Screen-Detected Cancers: A Nested Case Control Study from a Canadian Breast Screening Program. Laboratory Investigation. 2009;89:63A-4A.

117. Maygarden SJ, Desrosiers T, Wang L, Yankaskas BC. Comparison of pathologic features of screen detected (SDC) and interval (IC) breast cancers in African American (AAW) and white (WW) women. Modern Pathology. 2006;19:34A-A.

118. Huang C, Fann C, Hsu G, Ho M, Chang K, Chen S. A Population-Based Cross-Over Randomized Controlled Trial of Breast Cancer Screening with Alternate Mammography and Ultrasound for Women Aged 40 to 49 Years in Taiwan. 2010;69(24 Supplement).

119. Banik S, Rangayyan RM, Desautels JEL. Detection of architectural distortion in prior mammograms of interval-cancer cases with neural networks. Annual International Conference of the IEEE Engineering in Medicine and Biology Society IEEE Engineering in Medicine and Biology Society Annual International Conference. 2009;2009:6667-70.

120. Chlebowski RT, Anderson G, Pettinger M, Lane D, Langer RD, GillianMa. Estrogen plus progestin and breast cancer detection with mammography and breast biopsy. 2007.

121. Mills C, Sud A, Everall A, Chubb D, Lawrence SED, Kinnersley B, et al. Genetic landscape of interval and screen detected breast cancer. Npj Precision Oncology. 2024;8(1).

122. Hubbard RA, Su Y-R, Bowles EJA, Ichikawa L, Kerlikowske K, Lowry KP, et al. Predicting five-year interval second breast cancer risk in women with prior breast cancer. Jnci-Journal of the National Cancer Institute. 2024;116(6):929-37.

123. Freitas-Junior R, Rodrigues D, Correa R, Soares L. Breast cancer screening based on physical breast examination: ITABERAI randomized trial. Cancer research. 2024;84(9).

124. Dunn J, Donnelly P, Elbeltagi N, Marshall A, Thompson A, Audisio R, et al. Mammographic surveillance in early breast cancer patients aged 50 years or over: results of the Mammo-50 noninferiority trial of annual versus less frequent mammography. Cancer research. 2024;84(9).

125. de Munck L, Eijkelboom AH, Otten JDM, Broeders MJM, Siesling S. Method of primary breast cancer detection and the disease-free interval, adjusting for lead time. Jnci-Journal of the National Cancer Institute. 2024;116(3):370-8.

126. Ye Z, Nguyen TL, Dite GS, MacInnis RJ, Schmidt DF, Makalic E, et al. Causal relationships between breast cancer risk factors based on mammographic features. Breast Cancer Res. 2023;25(1):127.

127. Sendur HN, Sendur AB. The Distinction Between Interval and Missed Breast Cancer Requires Re-evaluation of Prior Imaging. Academic Radiology. 2023;30(12):3166-.

128. Razavi M, Bergerot CD, Philip EJ, Dale W. Association of time intervals in cancer screenings and older participants' characteristics, in a nationally representative sample. Journal of Geriatric Oncology. 2023;14(1).

129. Rahim A, Rasheed B, Adil SO, Naz N, Aslam N. Effective strategy to cope the pain and discomfort among women undergoing mammography-A randomized controlled trial. Pakistan journal of medical sciences. 2023;39(5):1422‐8.

130. Lee JM, Ichikawa LE, Wernli KJ, Bowles EJA, Specht JM, Kerlikowske K, et al. Impact of Surveillance Mammography Intervals Less Than One Year on Performance Measures in Women With a Personal History of Breast Cancer. Korean Journal of Radiology. 2023;24(8):729-38.

131. Kou K, Cameron J, Youl P, Pyke C, Chambers S, Dunn J, et al. Severity and risk factors of interval breast cancer in Queensland, Australia: a population-based study. Breast Cancer. 2023;30(3):466-77.

132. Khodayarian M, Mazloomy Mahmoodabad SS, Ali Morowati Sharifabad M, Lamyian M, Tavangar H. Investigating the Effect of Tailored Educational Program Based on Protection Motivation Theory on Mammography Adherence in Iranian Women. Journal of education and community health. 2023;10(3):152‐61.

133. Han HJ, Chu YC, Wang J, Lai YC, Tseng LM, Huang CC. Characteristics of breast cancers detected by screening mammography in Taiwan: a single institute's experience. BMC Womens Health. 2023;23(1):330.

134. Grigg J, Manning V, Lockie D, Giles M, Bell RJ, Stragalinos P, et al. A brief intervention for improving alcohol literacy and reducing harmful alcohol use by women attending a breast screening service: a randomised controlled trial. Medical journal of Australia. 2023;218(11):511‐9.

135. Goossens MM, Kellen E, Broeders MJM, Vandemaele E, Jacobs B, Martens P. The effect of a pre-scheduled appointment on attendance in a population-based mammography screening programme. European journal of public health. 2023;33(6):1122‐7.

136. Freitas V, Waterston ML, Olsen KO, Bubon O, Parker S, Baldassi B, et al. An Emerging Technology for Breast Cancer Detection - Preliminary Data of Breast Cancer Detection using Novel Low Dose Positron Emission Mammography. Cancer research. 2023;83(5).

137. Fitzpatrick P, Byrne H, Flanagan F, O'Doherty A, Connors A, Larke A, et al. Interval cancer audit and disclosure in breast screening programmes: An international survey. J Med Screen. 2023;30(1):36-41.

138. Eijkelboom AH, Larsen M, Siesling S, Nygård JF, Hofvind S, de Munck L. Prolonged screening interval due to the COVID-19 pandemic and its association with tumor characteristics and treatment; a register-based study from BreastScreen Norway. Prev Med. 2023;175:107723.

139. Claringbold L, Brennan ME, Lund H, El-Zaemey S, Houssami N, Wylie E. Reflections from Women with an Interval Breast Cancer Diagnosis: A Qualitative Analysis of Open Disclosure in the BreastScreen Western Australia Program. Asian Pac J Cancer Prev. 2023;24(2):633-9.

140. Çelik L, Güner DC, Özçağlayan Ö, Çubuk R, Arıbal ME. Diagnostic performance of two versions of an artificial intelligence system in interval breast cancer detection. Acta Radiol. 2023;64(11):2891-7.

141. Ambinder EB, Lee E, Nguyen DL, Gong AJ, Haken OJ, Visvanathan K. Interval Breast Cancers Versus Screen Detected Breast Cancers: A Retrospective Cohort Study. Acad Radiol. 2023;30 Suppl 2:S154-s60.

142. Hacek RT, Antoljak N, Erceg M. Development of a model for predicting risk from breast interval cancer in the female population in the Republic of Croatia. Neoplasma. 2022;69(3):708-22.

143. Groome PA, Webber C, Maxwell CJ, McClintock C, Seitz D, Mahar A, et al. Multiple Sclerosis and the Cancer Diagnosis Diagnostic Route, Cancer Stage, and the Diagnostic Interval in Breast and Colorectal Cancer. Neurology. 2022;98(18):E1798-E809.

144. Gordon PB. Breast Density and Risk of Interval Cancers. Canadian Association of Radiologists Journal. 2022;73(1):19-20.

145. Dreher N, Matthys M, Hadeler E, Shieh Y, Acerbi I, McAuley FM, et al. A case-case analysis of women with breast cancer: predictors of interval vs screen-detected cancer. Breast Cancer Research and Treatment. 2022;191(3):623-9.

146. Bucchi L, Ravaioli A, Baldacchini F, Giuliani O, Mancini S, Vattiato R, et al. Five-year annual incidence and clinico-molecular features of breast cancer after the last negative screening mammography at age 68-69. Eur Radiol. 2022;32(2):834-41.

147. Boyle T, Reintals M, Holmes A, Buckley E, Roder D. Interval cancers as related to frequency of recall to assessment in the South Australian population-based breast screening program: An exploratory study. Cancer Epidemiology. 2022;79.

148. Blackmore KM, Chiarelli AM, Mirea L, Mittmann N, Muradali D, Rabeneck L, et al. Annual Mammographic Screening Reduces the Risk of Interval or Higher Stage Invasive Breast Cancers Among Postmenopausal Women in the Ontario Breast Screening Program. Canadian Association of Radiologists Journal-Journal De L Association Canadienne Des Radiologistes. 2022;73(3):524-34.

149. Bertrand C, Bihan-Benjamin CL, Molinié F, Rogel A, Méric JB, Ifrah N, et al. Care pathway of women with interval breast cancer in 2016, based on medico-administrative data. Clin Breast Cancer. 2022;22(5):e718-e26.

150. Alabousi A, Patlas MN. Annual Mammographic Screening Reduces the Risk of Interval or Higher Stage Invasive Breast Cancers: Lessons for Today and Tomorrow. Canadian Association of Radiologists Journal-Journal De L Association Canadienne Des Radiologistes. 2022;73(3):446-7.

151. Yu H, Meng X, Chen H, Liu J, Gao W, Du L, et al. Predicting the Level of Tumor-Infiltrating Lymphocytes in Patients With Breast Cancer: usefulness of Mammographic Radiomics Features. Frontiers in oncology. 2021;11.

152. Wang S, Mao N, Duan S, Li Q, Li R, Jiang T, et al. Radiomic Analysis of Contrast-Enhanced Mammography With Different Image Types: classification of Breast Lesions. Frontiers in oncology. 2021;11.

153. van Ravesteyn NT, Schechter CB, Hampton JM, Alagoz O, van den Broek JJ, Kerlikowske K, et al. Trade-Offs Between Harms and Benefits of Different Breast Cancer Screening Intervals Among Low-Risk Women. Jnci-Journal of the National Cancer Institute. 2021;113(8):1017-26.

154. Tsuruda KM, Hovda T, Bhargava S, Veierød MB, Hofvind S. Survival among women diagnosed with screen-detected or interval breast cancer classified as true, minimal signs, or missed through an informed radiological review. Eur Radiol. 2021;31(5):2677-86.

155. Sardu C, Gatta G, Pieretti G, Viola L, Sacra C, Di Grezia G, et al. Pre-Menopausal Breast Fat Density Might Predict MACE During 10 Years of Follow-Up: the BRECARD Study. JACC Cardiovascular imaging. 2021;14(2):426‐38.

156. Park VY, Kim MJ, Kim GR, Yoon JH. Outcomes Following Negative Screening MRI Results in Korean Women with a Personal History of Breast Cancer: Implications for the Next MRI Interval. Radiology. 2021;300(2):303-11.

157. Monib S, Narula S, Breunung-Joshi N. Interval Breast Cancer Epidemiology, Radiology and Biological Characteristics. Indian Journal of Surgery. 2021;83(SUPPL 2):328-32.

158. McWilliams L, Woof VG, Donnelly LS, Howell A, Evans DG, French DP. Extending screening intervals for women at low risk of breast cancer: do they find it acceptable? Bmc Cancer. 2021;21(1).

159. Mao Z, Nystrom L, Jonsson H. Breast cancer screening with mammography in women aged 40-49 years: Impact of length of screening interval on effectiveness of the program. Journal of Medical Screening. 2021;28(2):200-6.

160. Mao N, Jiao Z, Duan S, Xu C, Xie H. Preoperative prediction of histologic grade in invasive breast cancer by using contrast-enhanced spectral mammography-based radiomics. Journal of X-ray science and technology. 2021;29(5):763‐72.

161. Mango VL, Heller SL. Beyond the <i>AJR</i>: "Trade-Offs Between Harms and Benefits of Different Breast Cancer Screening Intervals Among Low-Risk Women". American Journal of Roentgenology. 2021;217(3):770-.

162. Lynch T, Partridge A, Thompson A, Frank E, Pinto D, Collyar D, et al. Comparing an operation tomonitoring, with or without endocrine therapy (COMET): aprospective randomized trial for low-risk DCIS (AFT-25). Cancer research. 2021;81(4 SUPPL).

163. Kim GR, Cho N, Kim S-Y, Han W, Moon WK. Interval Cancers after Negative Supplemental Screening Breast MRI Results in Women with a Personal History of Breast Cancer. Radiology. 2021;300(2):314-23.

164. Hersch J, Barratt A, McGeechan K, Jansen J, Houssami N, Dhillon H, et al. Informing Women About Overdetection in Breast Cancer Screening: two-Year Outcomes From a Randomized Trial. Journal of the National Cancer Institute. 2021;113(11):1523‐30.

165. Frankhauser DE, Jovanovic-Talisman T, Lai L, Yee LD, Wang LV, Mahabal A, et al. Spatiotemporal strategies to identify aggressive biology in precancerous breast biopsies. WIREs Mech Dis. 2021;13(2):e1506.

166. Daniaux M, Gruber L, Santner W, Czech T, Knapp R. Interval breast cancer: Analysis of occurrence, subtypes and implications for breast cancer screening in a model region. Eur J Radiol. 2021;143:109905.

167. Chang JM. MRI Screening Interval in Women with a History of Breast Cancer. Radiology. 2021;300(2):312-3.

168. Burnside ES, Warren LM, Myles J, Wilkinson LS, Wallis MG, Patel M, et al. Quantitative breast density analysis to predict interval and node-positive cancers in pursuit of improved screening protocols: a case-control study. British Journal of Cancer. 2021;125(6):884-92.

169. Bucchi L, Ravaioli A, Baldacchini F, Giuliani O, Mancini S, Vattiato R, et al. Incidence of interval breast cancer among women aged 45-49 in an organised mammography screening setting. J Med Screen. 2021;28(2):207-9.

170. Aghamohammadi V, Salari-Moghaddam A, Benisi-Kohansal S, Taghavi M, Azadbakht L, Esmaillzadeh A. Adherence to the MIND Diet and Risk of Breast Cancer: a Case-control Study. Clinical breast cancer. 2021;21(3):e158‐e64.

171. MacInnes EG, Duffy SW, Simpson JA, Wallis MG, Turnbull AE, Wilkinson LS, et al. Radiological audit of interval breast cancers: Estimation of tumour growth rates. Breast. 2020;51:114-9.

172. Liu Q, Liu Z, Yong S, Jia K, Razmjooy N. Computer-aided breast cancer diagnosis based on image segmentation and interval analysis. Automatika. 2020;61(3):496-506.

173. Kaplan HG, Malmgren JA, Atwood MK. Breast cancer distant recurrence lead time interval by detection method in an institutional cohort. Bmc Cancer. 2020;20(1).

174. Irvin VL, Zhang Z, Simon MS, Chlebowski RT, Luoh S-W, Shadyab AH, et al. Comparison of Mortality Among Participants of Women's Health Initiative Trials With Screening-Detected Breast Cancers vs Interval Breast Cancers. Jama Network Open. 2020;3(6).

175. Hudson SM, Wilkinson LS, De Stavola BL, Dos-Santos-Silva I. Left-right breast asymmetry and risk of screen-detected and interval cancers in a large population-based screening population. British Journal of Radiology. 2020;93(1112).

176. Holen Å, Sebuødegård S, Waade GG, Aase H, Hopland NM, Pedersen K, et al. Screening at stationary versus mobile units in BreastScreen Norway. J Med Screen. 2020;27(1):31-9.

177. Henderson LM, O'Meara ES, Haas JS, Lee CI, Kerlikowske K, Sprague BL, et al. The Role of Social Determinants of Health in Self-Reported Access to Health Care Among Women Undergoing Screening Mammography. Journal of women's health (2002). 2020;29(11):1437‐46.

178. Duffy S, Vulkan D, Cuckle H, Parmar D, Sheikh S, Smith R, et al. Annual mammographic screening to reduce breast cancer mortality in women from age 40 years: long-term follow-up of the UK Age RCT. Health technology assessment (Winchester, England). 2020;24(55):1‐24.

179. Costanza ME, Luckmann R, Frisard C, White MJ, Cranos C. Comparing Telephone Counseling With Reminding to Promote On-Time Repeated Mammography: a Randomized Trial in a Cohort With 4 Years Follow-Up. Health education & behavior. 2020;47(1):37‐46.

180. Chlebowski RT, Anderson GL, Aragaki AK, Manson JE, Stefanick M, Pan K, et al. Long-term influence of estrogen plus progestin and estrogen alone use on breast cancer incidence: the women's health initiative randomized trials. Cancer research. 2020;80(4).

181. Cabioglu N, Gurdal SO, Kayhan A, Ozaydin N, Sahin C, Can O, et al. Poor Biological Factors and Prognosis of Interval Breast Cancers: Long-Term Results of Bahcesehir (Istanbul) Breast Cancer Screening Project in Turkey. JCO global oncology. 2020;6:1103-13.

182. Barros AF, Murta-Nascimento C, de Abdon CH, Nogueira DN, Cardoso Lopes EL, Dias A. Factors associated with time interval between the onset of symptoms and first medical visit in women with breast cancer. Cadernos De Saude Publica. 2020;36(2).

183. Azam S, Eriksson M, Sjolander A, Hellgren R, Gabrielson M, Czene K, et al. Mammographic Density Change and Risk of Breast Cancer. Journal of the National Cancer Institute. 2020;112(4):391‐9.

184. Abdolell M, Payne JI, Caines J, Tsuruda K, Barnes PJ, Talbot PJ, et al. Assessing breast cancer risk within the general screening population: developing a breast cancer risk model to identify higher risk women at mammographic screening. Eur Radiol. 2020;30(10):5417-26.

185. Yang GE, Kim E-K, Kim MJ, Moon HJ, Park VY, Yoon JH. Does Post-Biopsy Mammography at Short-Term Interval Contribute to Early Detection of Cancer in Patients Diagnosed with Benign-Concordant Microcalcifications on Stereotactic Biopsy? Iranian Journal of Radiology. 2019;16(3).

186. Tabár L, Dean PB, Chen TH, Yen AM, Chen SL, Fann JC, et al. The incidence of fatal breast cancer measures the increased effectiveness of therapy in women participating in mammography screening. Cancer. 2019;125(4):515‐23.

187. Strand F, Azavedo E, Hellgren R, Humphreys K, Eriksson M, Shepherd J, et al. Localized mammographic density is associated with interval cancer and large breast cancer: a nested case-control study. Breast Cancer Res. 2019;21(1):8.

188. Rannikko A, Kilpelainen T, Matikainen M, Kenttamies A, Petas A, Santti H, et al. A population-based randomized trial of early detection of clinically significant prostate cancer (ProScreen): pilot phase results. European urology, supplements. 2019;18(1):e1535‐e6.

189. Prange A, Bokhof B, Polzer P, Tio J, Radke I, Heidinger O, et al. Higher Detection Rates of Biologically Aggressive Breast Cancers in Mammography Screening than in the Biennial Interval. Rofo-Fortschritte Auf Dem Gebiet Der Rontgenstrahlen Und Der Bildgebenden Verfahren. 2019;191(2):130-6.

190. Mannu GS, Groen EJ, Wang Z, Schaapveld M, Lips EH, Chung M, et al. Reliability of preoperative breast biopsies showing ductal carcinoma in situ and implications for non-operative treatment: a cohort study. Breast cancer research and treatment. 2019;178(2):409‐18.

191. Lee Y, Lee HS, Ahn SH, Son BH, Kim J, Lee SB. Is asymptomatic surveillance after standard treatment beneficial? : a 10yr-survival analysis of recurrent breast cancer patients by detection method of recurrence. Cancer research. 2019;79(4).

192. Lee JS, Kim HA, Cho SH, Lee HB, Park MH, Jeong J, et al. Five-Year Overall Survival of Interval Breast Cancers is Better than Non- Interval Cancers from Korean Breast Cancer Registry. Asian Pac J Cancer Prev. 2019;20(6):1717-26.

193. Kerlikowske K, Sprague BL, Tosteson ANA, Wernli KJ, Rauscher GH, Johnson D, et al. Strategies to Identify Women at High Risk of Advanced Breast Cancer During Routine Screening for Discussion of Supplemental Imaging. JAMA Intern Med. 2019;179(9):1230-9.

194. Iotti V, Giorgi Rossi P, Nitrosi A, Ravaioli S, Vacondio R, Campari C, et al. Comparing two visualization protocols for tomosynthesis in screening: specificity and sensitivity of slabs versus planes plus slabs. European radiology. 2019;29(7):3802‐11.

195. Ho PJ, Bok CM, Ishak HMM, Lim LY, Liu J, Wong FY, et al. Factors associated with false-positive mammography at first screen in an Asian population. PloS one. 2019;14(3):e0213615.

196. Hinton B, Ma L, Mahmoudzadeh AP, Malkov S, Fan B, Greenwood H, et al. Derived mammographic masking measures based on simulated lesions predict the risk of interval cancer after controlling for known risk factors: a case-case analysis. Medical Physics. 2019;46(3):1309-16.

197. Hinton B, Ma L, Mahmoudzadeh AP, Malkov S, Fan B, Greenwood H, et al. Deep learning networks find unique mammographic differences in previous negative mammograms between interval and screen-detected cancers: a case-case study. Cancer Imaging. 2019;19.

198. Haas JS, Giess CS, Harris KA, Ansolabehere J, Kaplan CP. Randomized Trial of Personalized Breast Density and Breast Cancer Risk Notification. Journal of general internal medicine. 2019;34(4):591‐7.

199. Grassmann F, He W, Eriksson M, Gabrielson M, Hall P, Czene K. Interval breast cancer is associated with other types of tumors. Nat Commun. 2019;10(1):4648.

200. Cheasley D, Li N, Rowley SM, Elder K, Mann GB, Loi S, et al. Molecular comparison of interval and screen-detected breast cancers. Journal of Pathology. 2019;248(2):243-52.

201. Bhargava S, Akslen LA, Bukholm IRK, Hofvind S. Performance measures among non-immigrants and immigrants attending BreastScreen Norway: a population-based screening programme. Eur Radiol. 2019;29(9):4833-42.

202. Bakker MF, de Lange SV, Pijnappel RM, Mann RM, Peeters PHM, Monninkhof EM, et al. Supplemental MRI Screening for Women with Extremely Dense Breast Tissue. New England journal of medicine. 2019;381(22):2091‐102.

203. Aarts AMWM, Duffy SW, Geurts SME, Vulkan DP, Houssami N, Zappa M, et al. Towards evidence-based follow-up intervals for breast cancer survivors: Estimates of the preclinical detectable phase of contralateral second breast cancer. Breast. 2019;45:70-4.

204. You JK, Song MK, Kim MJ, Kim EK, Moon HJ, Youk JH, et al. Can Biannual Ultrasound Surveillance Detect Smaller Second Cancers or Detect Cancers Earlier in Patients with Breast Cancer History? Ultrasound in medicine & biology. 2018;44(7):1355‐63.

205. van Bommel RMG, Voogd AC, Nederend J, Setz-Pels W, Louwman MWJ, Strobbe LJ, et al. Incidence and tumour characteristics of bilateral and unilateral interval breast cancers at screening mammography. Breast. 2018;38:101-6.

206. van Bommel R, Lameijer JRC, Voogd AC, Nederend J, Louwman MWJ, Setz-Pels W, et al. Tumour characteristics of bilateral screen-detected cancers and bilateral interval cancers in women participating at biennial screening mammography. European Journal of Radiology. 2018;108:215-21.

207. Unger-Saldana K, Ventosa-Santaularia D, Miranda A, Verduzco-Bustos G. Barriers and Explanatory Mechanisms of Delays in the Patient and Diagnosis Intervals of Care for Breast Cancer in Mexico. Oncologist. 2018;23(4):440-53.

208. Strand F. Determinants of Interval Cancer and Tumor Size among Breast Cancer Screening Participants2018.

209. Slater JS, Parks MJ, Nelson CL, Hughes KD. The Efficacy of Direct Mail, Patient Navigation, and Incentives for Increasing Mammography and Colonoscopy in the Medicaid Population: a Randomized Controlled Trial. Cancer epidemiology, biomarkers & prevention. 2018;27(9):1047‐56.

210. Singh D, Miettinen J, Duffy S, Malila N, Pitkäniemi J, Anttila A. Association of symptoms and interval breast cancers in the mammography-screening programme: population-based matched cohort study. Br J Cancer. 2018;119(11):1428-35.

211. Sankatsing VDV, Fracheboud J, de Munck L, Broeders MJM, van Ravesteyn NT, Heijnsdijk EAM, et al. Detection and interval cancer rates during the transition from screen-film to digital mammography in population-based screening. Bmc Cancer. 2018;18.

212. Sala M, Domingo L, Louro J, Tora-Rocamora I, Bare M, Ferrer J, et al. Survival and Disease-Free Survival by Breast Density and Phenotype in Interval Breast Cancers. Cancer Epidemiology Biomarkers & Prevention. 2018;27(8):908-16.

213. Puvanesarajah S, Nyante SJ, Kuzmiak CM, Chen M, Tse C-K, Sun X, et al. PAM50 and Risk of Recurrence Scores for Interval Breast Cancers. Cancer Prevention Research. 2018;11(6):327-35.

214. Park VY, Kim E-K, Kim MJ, Moon HJ, Yoon JH. Breast magnetic resonance imaging for surveillance of women with a personal history of breast cancer: outcomes stratified by interval between definitive surgery and surveillance MR imaging. Bmc Cancer. 2018;18.

215. Nguyen TL, Aung YK, Li S, Nhut Ho T, Evans CF, Baglietto L, et al. Predicting interval and screen-detected breast cancers from mammographic density defined by different brightness thresholds. Breast Cancer Research. 2018;20.

216. Murphy DR, Meyer AND, Vaghani V, Russo E, Sittig DF, Wei L, et al. Electronic Triggers to Identify Delays in Follow-Up of Mammography: harnessing the Power of Big Data in Health Care. Journal of the American College of Radiology : JACR. 2018;15(2):287‐95.

217. Moshina N, Sebuødegård S, Lee CI, Akslen LA, Tsuruda KM, Elmore JG, et al. Automated Volumetric Analysis of Mammographic Density in a Screening Setting: Worse Outcomes for Women with Dense Breasts. Radiology. 2018;288(2):343-52.

218. Moon HJ, Kim MJ, Yoon JH, Kim E-K. Follow-up interval for probably benign breast lesions on screening ultrasound in women at average risk for breast cancer with dense breasts. Acta Radiologica. 2018;59(9):1045-50.

219. Mohd Mujar NM. Presentation, Diagnosis and Treatment of Breast Cancer amongst Women Attending Public Hospitals in Malaysia: The Time Intervals and Associated Factors to Delay2018.

220. Mireles-Aguilar T, Tamez-Salazar J, Munoz-Lozano JF, Lopez-Martinez EA, Romero C, Platas A, et al. <i>Alerta Rosa</i>: Novel Alert and Navigation Breast Cancer Program in Nuevo Leon, Mexico, for Reducing Health System Interval Delays. Oncologist. 2018;23(12):1461-6.

221. Lee JM, Abraham L, Lam DL, Buist DSM, Kerlikowske K, Miglioretti DL, et al. Cumulative Risk Distribution for Interval Invasive Second Breast Cancers After Negative Surveillance Mammography. Journal of Clinical Oncology. 2018;36(20):2070-+.

222. Kerlikowske K, Scott CG, Mahmoudzadeh AP, Ma L, Winham S, Jensen MR, et al. Automated and Clinical Breast Imaging Reporting and Data System Density Measures Predict Risk for Screen-Detected and Interval Cancers A Case-Control Study. Annals of Internal Medicine. 2018;168(11):757-+.

223. Jodal HC, Loberg M, Holme O, Adami H-O, Bretthauer M, Emilsson L, et al. Mortality From Postscreening (Interval) Colorectal Cancers Is Comparable to That From Cancer in Unscreened Patients-A Randomized Sigmoidoscopy Trial. Gastroenterology. 2018;155(6):1787-+.

224. Jiang L, Gilbert J, Langley H, Moineddin R, Groome PA. Breast cancer detection method, diagnostic interval and use of specialized diagnostic assessment units across Ontario, Canada. Health Promotion and Chronic Disease Prevention in Canada-Research Policy and Practice. 2018;38(10):358-67.

225. Hinton BJ. Developing Measures of Interval Breast Cancer Risk2018.

226. Defossez G, Quillet A, Ingrand P. Aggressive primary treatments with favourable 5-year survival for screen-interval breast cancers. Bmc Cancer. 2018;18.

227. Chan EK, Wilson C, Tyldesley S, Olivotto IA, Lai A, Sam J, et al. Signed family physician reminder letters to women overdue for screening mammography: a randomized clinical trial. Journal of medical screening. 2018;25(3):149‐54.

228. Cardoso de Almeida T, Marques de Mello L, Saraiva de Castro Mattos J, Soares da Silva A, Aparecido Nunes A. Evaluation of the impact of physical exercise in reducing pain in women undergoing mammography: a randomized clinical trial. Pain medicine (Malden, Mass). 2018;19(1):9‐15.

229. Burnside ES, Vulkan D, Blanks RG, Duffy SW. Association between Screening Mammography Recall Rate and Interval Cancers in the UK Breast Cancer Service Screening Program: A Cohort Study. Radiology. 2018;288(1):47-54.

230. Brück N, Koskivuo I, Boström P, Saunavaara J, Aaltonen R, Parkkola R. Preoperative Magnetic Resonance Imaging in Patients With Stage I Invasive Ductal Breast Cancer: a Prospective Randomized Study. Scandinavian journal of surgery. 2018;107(1):14‐22.

231. Yen AM-F, Wu WY-Y, Tabar L, Duffy SW, Smith RA, Chen H-H. Initiators and promoters for the occurrence of screen-detected breast cancer and the progression to clinically-detected interval breast cancer. Journal of Epidemiology. 2017;27(3):98-106.

232. Wernli KJ, Arao RF, Hubbard RA, Sprague BL, Alford-Teaster J, Haas JS, et al. Change in Breast Cancer Screening Intervals Since the 2009 USPSTF Guideline. Journal of Womens Health. 2017;26(8):820-7.

233. Wanders JOP, Holland K, Karssemeijer N, Peeters PHM, Veldhuis WB, Mann RM, et al. The effect of volumetric breast density on the risk of screen-detected and interval breast cancers: a cohort study. Breast Cancer Res. 2017;19(1):67.

234. van Bommel RMG, Weber R, Voogd AC, Nederend J, Louwman MWJ, Venderink D, et al. Interval breast cancer characteristics before, during and after the transition from screen-film to full-field digital screening mammography. BMC Cancer. 2017;17(1):315.

235. Tohno E. Implementation of ultrasonography as screening tool-after J-start in Japan. Ultrasound in medicine & biology. 2017;43:S16.

236. Timmermans L, Bleyen L, Bacher K, Van Herck K, Lemmens K, Van Ongeval C, et al. Screen-detected versus interval cancers: Effect of imaging modality and breast density in the Flemish Breast Cancer Screening Programme. European Radiology. 2017;27(9):3810-9.

237. Strand F, Humphreys K, Eriksson M, Li J, Andersson TM, Törnberg S, et al. Longitudinal fluctuation in mammographic percent density differentiates between interval and screen-detected breast cancer. Int J Cancer. 2017;140(1):34-40.

238. Scoggins M, Dogan B, Ma J, Wei W, Song JB, Candelaria RP, et al. Short breast MRI screening trial in women at highrisk for breast cancer. Add to Collection. Journal of clinical oncology. 2017;35(15).

239. Román M, Quintana MJ, Ferrer J, Sala M, Castells X. Cumulative risk of breast cancer screening outcomes according to the presence of previous benign breast disease and family history of breast cancer: supporting personalised screening. Br J Cancer. 2017;116(11):1480-5.

240. Rauscher GH, Dabbous F, Dolecek TA, Friedewald SM, Tossas-Milligan K, Macarol T, et al. Absence of an anticipated racial disparity in interval breast cancer within a large health care organization. Ann Epidemiol. 2017;27(10):654-8.

241. Ott OJ, Strnad V, Stillkrieg W, Uter W, Beckmann MW, Fietkau R. Accelerated partial breast irradiation with external beam radiotherapy : first results of the German phase 2 trial. Strahlentherapie und Onkologie. 2017;193(1):55‐61.

242. Moshina N, Sebuødegård S, Hofvind S. Is breast compression associated with breast cancer detection and other early performance measures in a population-based breast cancer screening program? Breast Cancer Res Treat. 2017;163(3):605-13.

243. Miglioretti DL, Ichikawa L, Smith RA, Buist DSM, Carney PA, Geller B, et al. Correlation Between Screening Mammography Interpretive Performance on a Test Set and Performance in Clinical Practice. Academic radiology. 2017;24(10):1256‐64.

244. Mennella S, Paparo F, Revelli M, Baccini P, Secondini L, Barbagallo S, et al. Magnetic resonance imaging of breast cancer: does the time interval between biopsy and MRI influence MRI-pathology discordance in lesion sizing? Acta Radiologica. 2017;58(7):800-8.

245. Lekanidi K, Dilks P, Suaris T, Kennett S, Purushothaman H. Breast screening: What can the interval cancer review teach us? Are we perhaps being a bit too hard on ourselves? Eur J Radiol. 2017;94:13-5.

246. Houssami N, Hunter K. The epidemiology, radiology and biological characteristics of interval breast cancers in population mammography screening. NPJ Breast Cancer. 2017;3:12.

247. Holt RE, Topps A, Lim YY, Gandhi A. Tomosynthesis as an alternative to magnetic resonance imaging (MRI) in assessing invasive lobular carcinoma (ILC) multifocality. Cancer research Conference: 39th annual CTRC-AACR san antonio breast cancer symposium United states. 2017;77(4 Supplement 1) (no pagination).

248. Delacour-Billon S, Mathieu-Wacquant AL, Campone M, Auffret N, Amossé S, Allioux C, et al. Short-term and long-term survival of interval breast cancers taking into account prognostic features. Cancer Causes Control. 2017;28(1):69-76.

249. Bellio G, Marion R, Giudici F, Kus S, Tonutti M, Zanconati F, et al. Interval Breast Cancer Versus Screen-Detected Cancer: Comparison of Clinicopathologic Characteristics in a Single-Center Analysis. Clin Breast Cancer. 2017;17(7):564-71.

250. Arleo EK, Monticciolo DL, Monsees B, McGinty G, Sickles EA. Persistent Untreated Screening-Detected Breast Cancer: An Argument Against Delaying Screening or Increasing the Interval Between Screenings. Journal of the American College of Radiology. 2017;14(7):863-7.

251. Youl PH, Aitken JF, Turrell G, Chambers SK, Dunn J, Pyke C, et al. The Impact of Rurality and Disadvantage on the Diagnostic Interval for Breast Cancer in a Large Population-Based Study of 3202 Women in Queensland, Australia. International journal of environmental research and public health. 2016;13(11).

252. Weber RJP, van Bommel RMG, Setz-Pels W, Voogd AC, Klompenhouwer EG, Louwman MW, et al. Type and Extent of Surgery for Screen-Detected and Interval Cancers at Blinded Versus Nonblinded Double-Reading in a Population-Based Screening Mammography Program. Annals of Surgical Oncology. 2016;23(12):3822-30.

253. Weber RJP, van Bommel RMG, Louwman MW, Nederend J, Voogd AC, Jansen FH, et al. Characteristics and prognosis of interval cancers after biennial screen-film or full-field digital screening mammography. Breast Cancer Research and Treatment. 2016;158(3):471-83.

254. Trentham-Dietz A, Kerlikowske K, Stout NK, Miglioretti DL, Schechter CB, Ergun MA, et al. Tailoring Breast Cancer Screening Intervals by Breast Density and Risk for Women Aged 50 Years or Older: Collaborative Modeling of Screening Outcomes. Annals of Internal Medicine. 2016;165(10):700-+.

255. Strand F, Humphreys K, Cheddad A, Tornberg S, Azavedo E, Shepherd J, et al. Novel mammographic image features differentiate between interval and screen-detected breast cancer: a case-case study. Breast Cancer Research. 2016;10.

256. Sripaiboonkij N, Thinkamrop B, Promthet S, Kannawat C, Tangcharoensathien V, Ansusing T, et al. Breast Cancer Detection Rate, Incidence, Prevalence and Interval Cancer-related Mammography Screening Times among Thai Women. Asian Pacific journal of cancer prevention : APJCP. 2016;17(8):4137-41.

257. Shieh Y, Eklund M, Esserman L. Detection of ductal carcinoma in situ and subsequent interval cancers. Bmj-British Medical Journal. 2016;352.

258. Salvagnini E, Bosmans H, Van Ongeval C, Van Steen A, Michielsen K, Cockmartin L, et al. Impact of compressed breast thickness and dose on lesion detectability in digital mammography: FROC study with simulated lesions in real mammograms. Medical physics. 2016;43(9):5104.

259. Pepe MS, Janes H, Li CI, Bossuyt PM, Feng Z, Hilden J. Early-Phase Studies of Biomarkers: What Target Sensitivity and Specificity Values Might Confer Clinical Utility? Clin Chem. 2016;62(5):737-42.

260. Ohuchi N, Suzuki A, Sobue T, Kawai M, Yamamoto S, Zheng YF, et al. Sensitivity and specificity of mammography and adjunctive ultrasonography to screen for breast cancer in the Japan Strategic Anti-cancer Randomized Trial (J-START): a randomised controlled trial. Lancet (london, england). 2016;387(10016):341‐8.

261. Oeffinger KC, Ford J, Moskowitz CS, Chou JF, Henderson TO, Hudson MM, et al. The EMPOWER study: promoting breast cancer screening-A randomized controlled trial (RCT) in the Childhood Cancer Survivor Study (CCSS). Journal of clinical oncology. 2016;34.

262. Massat NJ, Dibden A, Parmar D, Cuzick J, Sasieni PD, Duffy SW. Impact of Screening on Breast Cancer Mortality: the UK Program 20 Years On. Cancer epidemiology, biomarkers & prevention. 2016;25(3):455‐62.

263. Marshall JK, Mbah OM, Ford JG, Phelan-Emrick D, Ahmed S, Bone L, et al. Effect of Patient Navigation on Breast Cancer Screening Among African American Medicare Beneficiaries: a Randomized Controlled Trial. Journal of general internal medicine. 2016;31(1):68‐76.

264. Lee K, Kim H, Lee JH, Jeong H, Shin SA, Han T, et al. Retrospective observation on contribution and limitations of screening for breast cancer with mammography in Korea: detection rate of breast cancer and incidence rate of interval cancer of the breast. Bmc Womens Health. 2016;16.

265. Hsieh JC-F, Cramb SM, McGree JM, Dunn NAM, Baade PD, Mengersen KL. Does geographic location impact the survival differential between screen- and interval-detected breast cancers? Stochastic Environmental Research and Risk Assessment. 2016;30(1):155-65.

266. Houssami N. Digital breast tomosynthesis (3D-mammography) for screening women with dense breasts. Expert Rev Med Devices. 2016;13(6):515-7.

267. Goodrich ME, Weiss J, Onega T, Balch SL, Buist DSM, Kerlikowske K, et al. The Role of Preoperative Magnetic Resonance Imaging in the Assessment and Surgical Treatment of Interval and Screen-Detected Breast Cancer in Older Women. Breast Journal. 2016;22(6):616-22.

268. Duffy SW, Dibden A, Michalopoulos D, Offman J, Parmar D, Jenkins J, et al. Screen detection of ductal carcinoma in situ and subsequent incidence of invasive interval breast cancers: a retrospective population-based study. Lancet Oncology. 2016;17(1):109-14.

269. Choi WJ, Cha JH, Kim HH, Shin HJ, Chae EY. Analysis of prior mammography with negative result in women with interval breast cancer. Breast Cancer. 2016;23(4):583-9.

270. Best AL, Spencer SM, Friedman DB, Hall IJ, Billings D. The Influence of Spiritual Framing on African American Women's Mammography Intentions: a Randomized Trial. Journal of health communication. 2016;21(6):620‐8.

271. Berg CD. Breast Cancer Screening Interval: Risk Level May Matter. Annals of Internal Medicine. 2016;165(10):737-8.

272. O'Mahony JF, van Rosmalen J, Mushkudiani NA, Goudsmit F-W, Eijkemans MJC, Heijnsdijk EAM, et al. The Influence of Disease Risk on the Optimal Time Interval between Screens for the Early Detection of Cancer: A Mathematical Approach. Medical Decision Making. 2015;35(2):183-95.

273. O'Brien KM, Dwane F, Kelleher T, Sharp L, Comber H. Interval cancer rates in the Irish national breast screening programme. J Med Screen. 2015;22(3):136-43.

274. Meshkat B, Prichard RS, Al-Hilli Z, Bass GA, Quinn C, O'Doherty A, et al. A comparison of clinicalepathological characteristics between symptomatic and interval breast cancer. Breast. 2015;24(3):278-82.

275. Li J, Holm J, Bergh J, Eriksson M, Darabi H, Lindstrom LS, et al. Breast cancer genetic risk profile is differentially associated with interval and screen-detected breast cancers. Annals of Oncology. 2015;26(3):517-22.

276. Lee JM, Buist DSM, Houssami N, Dowling EC, Halpern EF, Gazelle GS, et al. Five-Year Risk for Interval-Invasive Second Breast Cancer. Jnci-Journal of the National Cancer Institute. 2015;107(7).

277. Knox M, O'Brien A, Szabó E, Smith CS, Fenlon HM, McNicholas MM, et al. Impact of full field digital mammography on the classification and mammographic characteristics of interval breast cancers. Eur J Radiol. 2015;84(6):1056-61.

278. Kerlikowske K, Zhu W, Tosteson ANA, Sprague BL, Tice JA, Lehman CD, et al. Identifying Women With Dense Breasts at High Risk for Interval Cancer A Cohort Study. Annals of Internal Medicine. 2015;162(10):673-+.

279. Jones BA, Epstein L, Genao I, Nunez-Smith M, Vila HS, Claus E, et al. Perceived control over health and history of mammography screening in Hispanic/Latino women living in the Northeast United States. Cancer epidemiology biomarkers and prevention. 2015;24(10).

280. Johnson JM, Johnson AK, O'Meara ES, Miglioretti DL, Geller BM, Hotaling EN, et al. Breast cancer detection with short-interval follow-up compared with return to annual screening in patients with benign stereotactic or US-guided breast biopsy results. Radiology. 2015;275(1):54‐60.

281. Holm J, Humphreys K, Li J, Ploner A, Cheddad A, Eriksson M, et al. Risk factors and tumor characteristics of interval cancers by mammographic density. J Clin Oncol. 2015;33(9):1030-7.

282. Henderson LM, Miglioretti DL, Kerlikowske K, Wernli KJ, Sprague BL, Lehman CD. Breast Cancer Characteristics Associated With Digital Versus Film-Screen Mammography for Screen-Detected and Interval Cancers. American Journal of Roentgenology. 2015;205(3):676-84.

283. Gummersbach E, in der Schmitten J, Mortsiefer A, Abholz HH, Wegscheider K, Pentzek M. Willingness to participate in mammography screening: a randomized controlled questionnaire study of responses to two patient information leaflets with different factual content. Deutsches Arzteblatt international. 2015;112(5):61‐8.

284. Emaus MJ, Bakker MF, Peeters PH, Loo CE, Mann RM, de Jong MD, et al. MR Imaging as an Additional Screening Modality for the Detection of Breast Cancer in Women Aged 50-75 Years with Extremely Dense Breasts: the DENSE Trial Study Design. Radiology. 2015;277(2):527‐37.

285. Bucchi L, Frigerio A, Zorzi M, Fedato C, Angiolucci G, Bernardi D, et al. Problems, solutions, and perspectives in the evaluation of interval cancers in Italian mammography screening programmes: a position paper from the Italian group for mammography screening (GISMa). Epidemiol Prev. 2015;39(3 Suppl 1):52-7.

286. Bare M, Tora N, Salas D, Sentis M, Ferrer J, Ibanez J, et al. Mammographic and clinical characteristics of different phenotypes of screen-detected and interval breast cancers in a nationwide screening program. Breast Cancer Research and Treatment. 2015;154(2):403-15.

287. Andersen SB, Tornberg S, Kilpelainen S, Lynge E, Njor SH, Von Euler-Chelpin M. Measuring the burden of interval cancers in long-standing screening mammography programmes. Journal of Medical Screening. 2015;22(2):83-92.

288. Urbschat I, Heidinger O. Determination of interval cancer rates in the German mammography screening program using population-based cancer registry data. Bundesgesundheitsblatt-Gesundheitsforschung-Gesundheitsschutz. 2014;57(1):68-75.

289. Taylor-Phillips S, Wallis MG, Parsons H, Dunn J, Stallard N, Campbell H, et al. Changing case Order to Optimise patterns of Performance in mammography Screening (CO-OPS): study protocol for a randomized controlled trial. Trials. 2014;15:17.

290. Simon MS, Wassertheil-Smoller S, Thomson CA, Ray RM, Hubbell FA, Lessin L, et al. Mammography interval and breast cancer mortality in women over the age of 75. Breast Cancer Research and Treatment. 2014;148(1):187-95.

291. Seetoh T, Siew WF, Koh A, Liau WF, Koh GC, Lee JJ, et al. Overcoming Barriers to Mammography Screening: a Quasi-randomised Pragmatic Trial in a Community-based Primary Care Setting. Annals of the Academy of Medicine, Singapore. 2014;43(12):588‐94.

292. Renart-Vicens G, Puig-Vives M, Albanell J, Castaner F, Ferrer J, Carreras M, et al. Evaluation of the interval cancer rate and its determinants on the Girona health region's early breast cancer detection program. Bmc Cancer. 2014;14.

293. Nederend J, Duijm LEM, Louwman MWJ, Roumen RMH, Jansen FH, Voogd AC. Trends in surgery for screen-detected and interval breast cancers in a national screening programme. British Journal of Surgery. 2014;101(8):949-58.

294. Nederend J, Duijm LEM, Louwman MWJ, Coebergh JW, Roumen RMH, Lohle PN, et al. Impact of the transition from screen-film to digital screening mammography on interval cancer characteristics and treatment - A population based study from the Netherlands. European Journal of Cancer. 2014;50(1):31-9.

295. Narod SA, Wall C, Baines C, Miller AB, Sun P. Impact of screening mammography on mortality from breast cancer before age 60 in women 40 to 49 years of age. Current oncology (Toronto, Ont). 2014;21(5):217‐21.

296. Miller AB, Wall C, Baines CJ, Sun P, To T, Narod SA. Twenty five year follow-up for breast cancer incidence and mortality of the Canadian National Breast Screening Study: randomised screening trial. BMJ (Clinical research ed). 2014;348:g366.

297. Kawai M, Suzuki A, Nishino Y, Ohnuki K, Ishida T, Amari M, et al. Effect of screening mammography on cumulative survival of Japanese women aged 40-69 years with breast cancer. Breast Cancer. 2014;21(5):542-9.

298. Ishida T, Suzuki A, Kawai M, Narikawa Y, Saito H, Yamamoto S, et al. A randomized controlled trial to verify the efficacy of the use of ultrasonography in breast cancer screening aged 40-49 (J-START): 76 196 women registered. Japanese journal of clinical oncology. 2014;44(2):134‐40.

299. Hofvind S, Skaane P, Elmore JG, Sebuødegård S, Hoff SR, Lee CI. Mammographic performance in a population-based screening program: before, during, and after the transition from screen-film to full-field digital mammography. Radiology. 2014;272(1):52-62.

300. Fontein DB, Charehbili A, Nortier JW, Meershoek-Klein Kranenbarg E, Kroep JR, Putter H, et al. Efficacy of six month neoadjuvant endocrine therapy in postmenopausal, hormone receptor-positive breast cancer patients--a phase II trial. European journal of cancer (Oxford, England : 1990). 2014;50(13):2190‐200.

301. Fong Y, Evans J, Brook D, Kenkre J, Jarvis P, Gower Thomas K. The incidence and 10-year survival of interval breast cancers in Wales. Clinical Radiology. 2014;69(4):E168-E72.

302. Domingo L, Salas D, Zubizarreta R, Bare M, Sarriugarte G, Barata T, et al. Tumor phenotype and breast density in distinct categories of interval cancer: results of population-based mammography screening in Spain. Breast Cancer Research. 2014;16(1).

303. Dibden A, Offman J, Parmar D, Jenkins J, Slater J, Binysh K, et al. Reduction in interval cancer rates following the introduction of two-view mammography in the UK breast screening programme. British Journal of Cancer. 2014;110(3):560-4.

304. Carbonaro LA, Azzarone A, Paskeh BB, Brambilla G, Brunelli S, Calori A, et al. Interval breast cancers: absolute and proportional incidence and blinded review in a community mammographic screening program. European journal of radiology. 2014;83(2):e84‐91.

305. Boyd NF, Huszti E, Melnichouk O, Martin LJ, Hislop G, Chiarelli A, et al. Mammographic features associated with interval breast cancers in screening programs. Breast Cancer Res. 2014;16(4):417.

306. Blanch J, Sala M, Ibanez J, Domingo L, Fernandez B, Otegi A, et al. Impact of Risk Factors on Different Interval Cancer Subtypes in a Population-Based Breast Cancer Screening Programme. Plos One. 2014;9(10).

307. Bento MJ, Goncalves G, Aguiar A, Antunes L, Veloso V, Rodrigues V. Clinicopathological differences between interval and screen-detected breast cancers diagnosed within a screening programme in Northern Portugal. Journal of Medical Screening. 2014;21(2):104-9.

308. Autier P, Boniol M, Smans M, Boyle P. Randomized trials on mammography screening and the left-to-nature design. Journal of clinical oncology. 2014;32(15).

309. Araujo MC, Lima RCF, de Souza RMCR. Interval symbolic feature extraction for thermography breast cancer detection. Expert Systems with Applications. 2014;41(15):6728-37.

310. Rafferty EA, Park JM, Philpotts LE, Poplack SP, Sumkin JH, Halpern EF, et al. Assessing radiologist performance using combined digital mammography and breast tomosynthesis compared with digital mammography alone: results of a multicenter, multireader trial. Radiology. 2013;266(1):104‐13.

311. Prieto Garcia MA, Delgado Sevillano R, Baldo Sierra C, Gonzalez Diaz E, Lopez Secades A, Llavona Amor JA, et al. Classification and characteristics of interval cancers in the Principality of Asturias's Breast Cancer Screening Program. Radiologia. 2013;55(5):408-15.

312. Paci E, Mantellini P, Giorgi Rossi P, Falini P, Puliti D. Tailored Breast Screening Trial (TBST). Epidemiologia e prevenzione. 2013;37(4‐5):317‐27.

313. Houssami N, Abraham LA, Kerlikowske K, Buist DS, Irwig L, Lee J, et al. Risk factors for second screen-detected or interval breast cancers in women with a personal history of breast cancer participating in mammography screening. Cancer Epidemiol Biomarkers Prev. 2013;22(5):946-61.

314. Hoff SR. Breast Cancer: Missed Interval and Screening-detected Cancer at Full-Field Digital Mammography and Screen-Film Mammography-Results from a Retrospective Review (vol 264, pg 378, 2012). Radiology. 2013;266(1):367-.

315. Eriksson L, Czene K, Rosenberg LU, Tornberg S, Humphreys K, Hall P. Mammographic density and survival in interval breast cancers. Breast Cancer Research. 2013;15(3).

316. Duffy SW, Mackay J, Thomas S, Anderson E, Chen TH, Ellis I, et al. Evaluation of mammographic surveillance services in women aged 40-49 years with a moderate family history of breast cancer: a single-arm cohort study. Health technology assessment (Winchester, England). 2013;17(11):vii‐xiv, 1‐95.

317. Domingo L, Blanch J, Servitja S, Maria Corominas J, Murta-Nascimento C, Rueda A, et al. Aggressiveness features and outcomes of true interval cancers: comparison between screen-detected and symptom-detected cancers. European Journal of Cancer Prevention. 2013;22(1):21-8.

318. Dittus K, Geller B, Weaver DL, Kerlikowske K, Zhu W, Hubbard R, et al. Impact of Mammography Screening Interval on Breast Cancer Diagnosis by Menopausal Status and BMI. Journal of General Internal Medicine. 2013;28(11):1454-62.

319. De Koning H. The cost-effectiveness of cancer screening. European journal of cancer. 2013;49:S21.

320. Cappello NM. Decade of 'normal' mammography reports--the happygram. J Am Coll Radiol. 2013;10(12):903-8.

321. Wang JH, Schwartz MD, Brown RL, Maxwell AE, Lee MM, Adams IF, et al. Results of a randomized controlled trial testing the efficacy of a culturally targeted and a generic video on mammography screening among chinese-american immigrants. Cancer epidemiology, biomarkers & prevention. 2012;21(11):1923‐32.

322. Speiser D. MRI Screening in brca mutation carriers - the best alternative to prophylactic surgery? Breast care (Basel, Switzerland). 2012;7(6):503‐4.

323. Solbjør M, Skolbekken JA, Sætnan AR, Hagen AI, Forsmo S. Could screening participation bias symptom interpretation? An interview study on women's interpretations of and responses to cancer symptoms between mammography screening rounds. BMJ Open. 2012;2(6).

324. Solbjor M, Skolbekken J-A, Saetnan AR, Hagen AI, Forsmo S. Mammography screening and trust: The case of interval breast cancer. Social Science & Medicine. 2012;75(10):1746-52.

325. Peplonska B, Bukowska A, Sobala W, Reszka E, Gromadzinska J, Wasowicz W, et al. Rotating night shift work and mammographic density. Cancer epidemiology, biomarkers & prevention. 2012;21(7):1028‐37.

326. Pagán JA, Brown CJ, Asch DA, Armstrong K, Bastida E, Guerra C. Health literacy and breast cancer screening among Mexican American women in South Texas. Journal of cancer education. 2012;27(1):132‐7.

327. McLaughlin JM, Anderson RT, Ferketich AK, Seiber EE, Balkrishnan R, Paskett ED. Effect on Survival of Longer Intervals Between Confirmed Diagnosis and Treatment Initiation Among Low-Income Women With Breast Cancer. Journal of Clinical Oncology. 2012;30(36):4493-500.

328. Lin C, Buxton MB, Moore D, Krontiras H, Carey L, DeMichele A, et al. Locally advanced breast cancers are more likely to present as Interval Cancers: results from the I-SPY 1 TRIAL (CALGB 150007/150012, ACRIN 6657, InterSPORE Trial). Breast Cancer Research and Treatment. 2012;132(3):871-9.

329. Kalager M, Tamimi RM, Bretthauer M, Adami H-O. Prognosis in women with interval breast cancer: population based observational cohort study. Bmj-British Medical Journal. 2012;345.

330. Ishikawa Y, Hirai K, Saito H, Fukuyoshi J, Yonekura A, Harada K, et al. Cost-effectiveness of a tailored intervention designed to increase breast cancer screening among a non-adherent population: a randomized controlled trial. BMC public health. 2012;12:760.

331. Hymas RV, Gaffney DK, Parkinson BT, Belnap TW, Sause WT. Is Short-Interval Mammography Necessary After Breast Conservation Surgery and Radiation Treatment in Breast Cancer Patients? International Journal of Radiation Oncology Biology Physics. 2012;83(2):519-24.

332. Heidinger O, Batzler WU, Krieg V, Weigel S, Biesheuvel C, Heindel W, et al. The Incidence of Interval Cancers in the German Mammography Screening Program Results From the Population-Based Cancer Registry in North Rhine-Westphalia. Deutsches Arzteblatt International. 2012;109(46):781-U31.

333. Emaus M, Veldhuis W, Bakker M, Monninkhof E, Karssemeijer N, Van Den Bosch M, et al. Design of the DENSE trial: MRI as an additional screening modality to detect breast cancer in women aged 50-75 years with extremely dense breasts. Cancer prevention research (Philadelphia, Pa). 2012;5(11).

334. Crandall CJ, Aragaki AK, Cauley JA, McTiernan A, Manson JE, Anderson G, et al. Breast tenderness and breast cancer risk in the estrogen plus progestin and estrogen-alone women's health initiative clinical trials. Breast cancer research and treatment. 2012;132(1):275‐85.

335. Carney PA, Abraham L, Cook A, Feig SA, Sickles EA, Miglioretti DL, et al. Impact of an educational intervention designed to reduce unnecessary recall during screening mammography. Academic radiology. 2012;19(9):1114‐20.

336. Berg WA, Zhang Z, Lehrer D, Jong RA, Pisano ED, Barr RG, et al. Detection of breast cancer with addition of annual screening ultrasound or a single screening MRI to mammography in women with elevated breast cancer risk. JAMA. 2012;307(13):1394‐404.

337. Rayson D, Payne JI, Abdolell M, Barnes PJ, MacIntosh RF, Foley T, et al. Comparison of Clinical-Pathologic Characteristics and Outcomes of True Interval and Screen-Detected Invasive Breast Cancer Among Participants of a Canadian Breast Screening Program: A Nested Case-Control Study. Clinical Breast Cancer. 2011;11(1):27-32.

338. Parvinen I, Chiu S, Pylkkanen L, Klemi P, Immonen-Raiha P, Kauhava L, et al. Effects of annual vs triennial mammography interval on breast cancer incidence and mortality in ages 40-49 in Finland. British Journal of Cancer. 2011;105(9):1388-91.

339. Nuño T, Castle PE, Harris R, Estrada A, García F. Breast and cervical cancer screening utilization among hispanic women living near the United States-Mexico border. Journal of women's health (2002). 2011;20(5):685‐93.

340. Lowery JT, Byers T, Hokanson JE, Kittelson J, Lewin J, Risendal B, et al. Complementary approaches to assessing risk factors for interval breast cancer. Cancer Causes Control. 2011;22(1):23-31.

341. Lairson DR, Chan W, Chang YC, del Junco DJ, Vernon SW. Cost-effectiveness of targeted versus tailored interventions to promote mammography screening among women military veterans in the United States. Evaluation and program planning. 2011;34(2):97‐104.

342. Hoff SR, Samset JH, Abrahamsen A-L, Vigeland E, Klepp O, Hofvind S. Missed and True Interval and Screen-detected Breast Cancers in a Population Based Screening Program. Academic Radiology. 2011;18(4):454-60.

343. Dinkelspiel H, Flanagan T, Kinney W, Fetterman B, Lorey T, Castle P. Consequences of lengthening cervical cancer screening intervals on mammography rates. Gynecologic Oncology. 2011;121(1):S106-S7.

344. Corsetti V, Houssami N, Ghirardi M, Ferrari A, Speziani M, Bellarosa S, et al. Evidence of the effect of adjunct ultrasound screening in women with mammography-negative dense breasts: Interval breast cancers at 1 year follow-up. European Journal of Cancer. 2011;47(7):1021-6.

345. Chlebowski R. Re: Breast Cancer Risk in Relation to the Interval Between Menopause and Starting Hormone Therapy Response. Journal of the National Cancer Institute. 2011;103(13):1069-70.

346. Cardarelli K, Jackson R, Martin M, Linnear K, Lopez R, Senteio C, et al. Community-based participatory approach to reduce breast cancer disparities in South Dallas. Progress in community health partnerships : research, education, and action. 2011;5(4):375‐85.

347. Bennett RL, Sellars SJ, Moss SM. Interval cancers in the NHS breast cancer screening programme in England, Wales and Northern Ireland. British Journal of Cancer. 2011;104(4):571-7.

348. Bennett ML, Welman CJ, Celliers LM. How reassuring is a normal breast ultrasound in assessment of a screen-detected mammographic abnormality? A review of interval cancers after assessment that included ultrasound evaluation. Clin Radiol. 2011;66(10):928-39.

349. Banik S. Computer-aided Detection of Architectural Distortion in Prior Mammograms of Interval-cancer Cases2011.

350. Wu JC-Y, Hakama M, Anttila A, Yen AM-F, Malila N, Sarkeala T, et al. Estimation of natural history parameters of breast cancer based on non-randomized organized screening data: subsidiary analysis of effects of inter-screening interval, sensitivity, and attendance rate on reduction of advanced cancer. Breast Cancer Research and Treatment. 2010;122(2):553-66.

351. Woolcott CG, Courneya KS, Boyd NF, Yaffe MJ, Terry T, McTiernan A, et al. Mammographic density change with 1 year of aerobic exercise among postmenopausal women: a randomized controlled trial. Cancer epidemiology, biomarkers & prevention. 2010;19(4):1112‐21.

352. Törnberg S, Kemetli L, Ascunce N, Hofvind S, Anttila A, Sèradour B, et al. A pooled analysis of interval cancer rates in six European countries. Eur J Cancer Prev. 2010;19(2):87-93.

353. Russell KM, Champion VL, Monahan PO, Millon-Underwood S, Zhao Q, Spacey N, et al. Randomized trial of a lay health advisor and computer intervention to increase mammography screening in African American women. Cancer epidemiology, biomarkers & prevention. 2010;19(1):201‐10.

354. Ravaioli A, Foca F, Colamartini A, Falcini F, Naldoni C, Finarelli AC, et al. Incidence, detection, and tumour stage of breast cancer in a cohort of Italian women with negative screening mammography report recommending early (short-interval) rescreen. BMC Med. 2010;8:11.

355. Rangayyan RM, Banik S, Desautels JEL. Computer-Aided Detection of Architectural Distortion in Prior Mammograms of Interval Cancer. Journal of Digital Imaging. 2010;23(5):611-31.

356. Kuhl C, Weigel S, Schrading S, Arand B, Bieling H, Konig R, et al. Prospective multicenter cohort study to refine management recommendations for women at elevated familial risk of breast cancer: the EVA trial. Journal of clinical oncology. 2010;28(9):1450‐7.

357. Kingston N, Thomas I, Johns L, Moss S. Assessing the amount of unscheduled screening ("contamination") in the control arm of the UK "Age" Trial. Cancer epidemiology, biomarkers & prevention. 2010;19(4):1132‐6.

358. Domingo L, Sala M, Servitja S, Corominas JM, Ferrer F, Martínez J, et al. Phenotypic characterization and risk factors for interval breast cancers in a population-based breast cancer screening program in Barcelona, Spain. Cancer Causes Control. 2010;21(8):1155-64.

359. Chlebowski RT, Anderson G, Manson JE, Pettinger M, Yasmeen S, Lane D, et al. Estrogen alone in postmenopausal women and breast cancer detection by means of mammography and breast biopsy. Journal of clinical oncology. 2010;28(16):2690‐7.

360. Chiu SY, Duffy S, Yen AM, Tabár L, Smith RA, Chen HH. Effect of baseline breast density on breast cancer incidence, stage, mortality, and screening parameters: 25-year follow-up of a Swedish mammographic screening. Cancer epidemiology, biomarkers & prevention. 2010;19(5):1219‐28.

361. Caumo F, Vecchiato F, Strabbioli M, Zorzi M, Baracco S, Ciatto S. Interval cancers in breast cancer screening: comparison of stage and biological characteristics with screen-detected cancers or incident cancers in the absence of screening. Tumori Journal. 2010;96(2):198-201.

362. Vujovic O, Yu E, Cherian A, Perera F, Dar AR, Stitt L, et al. EFFECT OF INTERVAL TO DEFINITIVE BREAST SURGERY ON CLINICAL PRESENTATION AND SURVIVAL IN EARLY-STAGE INVASIVE BREAST CANCER. International Journal of Radiation Oncology Biology Physics. 2009;75(3):771-4.

363. Stewart SL, Rakowski W, Pasick RJ. Behavioral constructs and mammography in five ethnic groups. Health education & behavior. 2009;36(5 Suppl):36S‐54S.

364. Sperati A, Pannozzo F, Rossi PG, Baiocchi D, Barca A, Borgia P. Validating regional Hospital Information System data through comparison with a local cancer register to identify interval cancers of a breast screening program. European Journal of Cancer Prevention. 2009;18(3):212-5.

365. Seigneurin A, Exbrayat C, Labarère J, Colonna M. Comparison of interval breast cancer rates for two-versus single-view screening mammography: a population-based study. Breast. 2009;18(5):284-8.

366. Rejnmark L, Tietze A, Vestergaard P, Buhl L, Lehbrink M, Heickendorff L, et al. Reduced pre-diagnostic 25-hydroxyvitamin D levels in women with breast cancer. Bone. 2009;44:S166.

367. Park CC, Rembert J, Chew K, Moore D, Kerlikowske K. High mammographic breast density is independent predictor of local but not distant recurrence after lumpectomy and radiotherapy for invasive breast cancer. International journal of radiation oncology, biology, physics. 2009;73(1):75‐9.

368. Nelson HD, Tyne K, Naik A, Bougatsos C, Chan BK, Humphrey L. Screening for breast cancer: an update for the U.S. Preventive Services Task Force. Annals of internal medicine. 2009;151(10):727‐37, W237‐42.

369. Malich A, Schmidt S, Fischer DR, Facius M, Kaiser WA. The performance of computer-aided detection when analyzing prior mammograms of newly detected breast cancers with special focus on the time interval from initial imaging to detection. European Journal of Radiology. 2009;69(3):574-8.

370. Hofvind S, Yankaskas BC, Bulliard J-L, Klabunde CN, Fracheboud J. Comparing interval breast cancer rates in Norway and North Carolina: results and challenges. Journal of Medical Screening. 2009;16(3):131-9.

371. Crandall CJ, Aragaki AK, Chlebowski RT, McTiernan A, Anderson G, Hendrix SL, et al. New-onset breast tenderness after initiation of estrogen plus progestin therapy and breast cancer risk. Archives of internal medicine. 2009;169(18):1684‐91.

372. Caumo F, Vecchiato F, Pellegrini M, Vettorazzi M, Ciatto S, Montemezzi S. Analysis of interval cancers observed in an Italian mammography screening programme (2000-2006). Radiologia Medica. 2009;114(6):907-14.

373. Bordas P, Jonsson H, Nystrom L, Lenner P. Interval cancer incidence and episode sensitivity in the Norrbotten Mammography Screening Programme, Sweden. Journal of Medical Screening. 2009;16(1):39-45.

374. Beaver K, Tysver-Robinson D, Campbell M, Twomey M, Williamson S, Hindley A, et al. Comparing hospital and telephone follow-up after treatment for breast cancer: randomised equivalence trial. BMJ (Clinical research ed). 2009;338:a3147.

375. Tchou J, Greshock J, Bergey MR, Sonnad SS, Sargen M, Weinstein S, et al. Method of primary tumor detection as a risk factor for local and distant recurrence after breast-conservation treatment for early-stage breast cancer. Clin Breast Cancer. 2008;8(2):143-8.

376. Suzuki A, Kuriyama S, Kawai M, Amari M, Takeda M, Ishida T, et al. Age-specific interval breast cancers in Japan: estimation of the proper sensitivity of screening using a population-based cancer registry. Cancer Science. 2008;99(11):2264-7.

377. Shen Y, Costantino JP, Qin J. Tamoxifen chemoprevention treatment and time to first diagnosis of estrogen receptor-negative breast cancer. Journal of the National Cancer Institute. 2008;100(20):1448‐53.

378. Lin K, Eradat J, Mehta NH, Bent C, Lee SP, Apple SK, et al. IS A SHORT-INTERVAL POSTRADIATION MAMMOGRAM NECESSARY AFTER CONSERVATIVE SURGERY AND RADIATION IN BREAST CANCER? International Journal of Radiation Oncology Biology Physics. 2008;72(4):1041-7.

379. Kellen E, Putte GV, Van Steen A, Cloes E, Lousbergh D, Buntinx F, et al. Interval cancers in the beginning years of the breast cancer screening programme in the Belgian province of Limburg. Acta Clinica Belgica. 2008;63(3):179-84.

380. Jandorf L, Bursac Z, Pulley L, Trevino M, Castillo A, Erwin DO. Breast and cervical cancer screening among Latinas attending culturally specific educational programs. Progress in community health partnerships : research, education, and action. 2008;2(3):195‐204.

381. Hofvind S, Geller B, Skaane P. Mammographic features and histopathological findings of interval breast cancers. Acta Radiol. 2008;49(9):975-81.

382. Daguet E, Malhaire C, Hardit C, Athanasiou A, El KC, Thibault F, et al. MR breast screening in patients with genetic mutation. Journal de radiologie. 2008;Elsevier Masson SAS. 89(6):783‐90.

383. Ciatto S, Naldoni C, Ponti A, Giordano L, Giorgi D, Frigerio A, et al. Interval cancers as indicators of performance in screening programmes. Epidemiologia & Prevenzione. 2008;32(2):93-8.

384. Bucchi L, Ravaioli A, Foca F, Colamartini A, Falcini F, Naldoni C, et al. Incidence of interval breast cancers after 650,000 negative mammographies in 13 Italian health districts. Journal of Medical Screening. 2008;15(1):30-5.

385. Bucchi L, Puliti D, Ravaioli A, Cortesi L, De Lisi V, Falcini F, et al. Breast screening: Axillary lymph node status of interval cancers by interval year. Breast. 2008;17(5):477-83.

386. Berg WA, Blume JD, Cormack JB, Mendelson EB, Lehrer D, Böhm-Vélez M, et al. Combined screening with ultrasound and mammography vs mammography alone in women at elevated risk of breast cancer. JAMA. 2008;299(18):2151‐63.

387. Skaane P, Hofvind S, Skjennald A. Randomized trial of screen-film versus full-field digital mammography with soft-copy reading in population-based screening program: follow-up and final results of Oslo II study. Radiology. 2007;244(3):708‐17.

388. Porter GJR, Evans AJ, Cornford EJ, Burrell HC, James JJ, Lee AHS, et al. Influence of mammographic parenchymal pattern in screening-detected and interval invasive breast cancers on pathologic features, mammographic features, and patient survival. American Journal of Roentgenology. 2007;188(3):676-83.

389. Porter GJR, Evans AJ, Burrell HC, Lee AHS, Chakrabarti J. NHSBSP type 1 interval cancers: a scientifically valid grouping? Clinical Radiology. 2007;62(3):262-7.

390. Mishra SI, Bastani R, Crespi CM, Chang LC, Luce PH, Baquet CR. Results of a randomized trial to increase mammogram usage among Samoan women. Cancer epidemiology, biomarkers & prevention. 2007;16(12):2594‐604.

391. Han PK, Kobrin SC, Klein WM, Davis WW, Stefanek M, Taplin SH. Perceived ambiguity about screening mammography recommendations: association with future mammography uptake and perceptions. Cancer epidemiology, biomarkers & prevention. 2007;16(3):458‐66.

392. Hagen AI, Kvistad KA, Maehle L, Holmen MM, Aase H, Styr B, et al. Sensitivity of MRI versus conventional screening in the diagnosis of BRCA-associated breast cancer in a national prospective series. Breast (Edinburgh, Scotland). 2007;16(4):367‐74.

393. Goel A, Littenberg B, Burack RC. The association between the pre-diagnosis mammography screening interval and advanced breast cancer. Breast Cancer Research and Treatment. 2007;102(3):339-45.

394. Evans AJ, Kutt E, Record C, Waller M, Bobrow L, Moss S. Radiological and pathological findings of interval cancers in a multi-centre, randomized, controlled trial of mammographic screening in women from age 40-41 years. Clinical Radiology. 2007;62(4):348-52.

395. Dietrich AJ, Tobin JN, Cassells A, Robinson CM, Reh M, Romero KA, et al. Translation of an efficacious cancer-screening intervention to women enrolled in a Medicaid managed care organization. Annals of family medicine. 2007;5(4):320‐7.

396. Ciatto S, Catarzi S, Lamberini MP, Risso G, Saguatti G, Abbattista T, et al. Interval breast cancers in screening: The effect of mammography review method on classification. Breast. 2007;16(6):646-52.

397. Chamot E, Charvet AI, Perneger TV. Who gets screened, and where: a comparison of organised and opportunistic mammography screening in Geneva, Switzerland. European journal of cancer. 2007;43(3):576‐84.

398. Bordas P, Jonsson H, Nystroem L, Lenner P. Survival from invasive breast cancer among interval cases in the mammography screening programmes of northern Sweden. Breast. 2007;16(1):47-54.

399. Zackrisson S, Andersson I, Janzon L, Manjer J, Garne JP. Rate of over-diagnosis of breast cancer 15 years after end of Malmo mammographic screening trial: follow-up study. BMJ (Clinical research ed). 2006;332(7543):689‐92.

400. Zackrisson S. Breast cancer screening in an urban Swedish population: Aspects of non-attendance, interval cancers and over-diagnosis2006.

401. Vettorazzi M, Stocco C, Chirico A, Recanatini S, Saccon S, Mariotto R, et al. Quality control of mammography screening in the Veneto region. Evaluation of four programs at a Local Health Unit level - Analysis of the frequency and diagnostic pattern of interval cancers. Tumori Journal. 2006;92(1):1-5.

402. Stefanick ML, Anderson GL, Margolis KL, Hendrix SL, Rodabough RJ, Paskett ED, et al. Effects of conjugated equine estrogens on breast cancer and mammography screening in postmenopausal women with hysterectomy. JAMA. 2006;295(14):1647‐57.

403. Porter GJ, Evans AJ, Burrell HC, Lee AH, Ellis IO, Chakrabarti J. Interval breast cancers: prognostic features and survival by subtype and time since screening. J Med Screen. 2006;13(3):115-22.

404. Paskett E, Tatum C, Rushing J, Michielutte R, Bell R, Long Foley K, et al. Randomized trial of an intervention to improve mammography utilization among a triracial rural population of women. Journal of the National Cancer Institute. 2006;98(17):1226‐37.

405. Hofvind S, Møller B, Thoresen S, Ursin G. Use of hormone therapy and risk of breast cancer detected at screening and between mammographic screens. Int J Cancer. 2006;118(12):3112-7.

406. Hofvind S, Bjurstam N, Sørum R, Bjørndal H, Thoresen S, Skaane P. Number and characteristics of breast cancer cases diagnosed in four periods in the screening interval of a biennial population-based screening programme. J Med Screen. 2006;13(4):192-6.

407. Emdin SO, Granstrand B, Ringberg A, Sandelin K, Arnesson LG, Nordgren H, et al. SweDCIS: radiotherapy after sector resection for ductal carcinoma in situ of the breast. Results of a randomised trial in a population offered mammography screening. Acta oncologica (Stockholm, Sweden). 2006;45(5):536‐43.

408. Bulliard J-L, Sasieni P, Klabunde C, De Landtsheer J-P, Yankaskas BC, Fracheboud J. Methodological issues in international comparison of interval breast cancers. International Journal of Cancer. 2006;119(5):1158-63.

409. Anonymous. Digital mammography more sensitive for younger women. Journal of the National Medical Association. 2006;98(1):101.

410. Törnberg S, Codd M, Rodrigues V, Segnan N, Ponti A. Ascertainment and evaluation of interval cancers in population-based mammography screening programmes:: a collaborative study in four European centres. Journal of Medical Screening. 2005;12(1):43-9.

411. Shen Y, Yang Y, Inoue LY, Munsell MF, Miller AB, Berry DA. Role of detection method in predicting breast cancer survival: analysis of randomized screening trials. Journal of the National Cancer Institute. 2005;97(16):1195‐203.

412. Moss S, Waller M, Anderson TJ, Cuckle H. Randomised controlled trial of mammographic screening in women from age 40: predicted mortality based on surrogate outcome measures. British journal of cancer. 2005;92(5):955‐60.

413. McTiernan A, Martin CF, Peck JD, Aragaki AK, Chlebowski RT, Pisano ED, et al. Estrogen-plus-progestin use and mammographic density in postmenopausal women: women's Health Initiative randomized trial. Journal of the National Cancer Institute. 2005;97(18):1366‐76.

414. McAlearney AS, Reeves KW, Tatum C, Paskett ED. Perceptions of insurance coverage for screening mammography among women in need of screening. Cancer. 2005;103(12):2473‐80.

415. Lowery JT. A study of the determinants, characteristics and outcomes of interval breast cancer2005.

416. Kerlikowske K, Smith-Bindman R, Abraham LA, Lehman CD, Yankaskas BC, Ballard-Barbash R, et al. Breast cancer yield for screening mammographic examinations with recommendation for short-interval follow-up. Radiology. 2005;234(3):684-92.

417. Collett K, Stefansson IM, Eide J, Braaten A, Wang H, Eide GE, et al. A basal epithelial phenotype is more frequent in interval breast cancers compared with screen detected tumors. Cancer Epidemiology Biomarkers & Prevention. 2005;14(5):1108-12.

418. Carney PA, Harwood BG, Greene MA, Goodrich ME. Impact of a telephone counseling intervention on transitions in stage of change and adherence to interval mammography screening (United States). Cancer causes & control. 2005;16(7):799‐807.

419. Burani R, Caimi F, Maggioni C, Marinoni G, Pellizzoni R, Pirola ME, et al. Quality assessment of the mammographic screening programme in the Azienda Sanitaria locale Provincia Milano I - Analysis of interval cancers and discussion of possible causes of diagnostic error. Radiologia Medica. 2005;109(3):260-7.

420. Abood DA, Black DR, Coster DC. Loss-framed minimal intervention increases mammography use. Women's health issues. 2005;15(6):258‐64.

421. Taylor R, Page A, Bampton D, Estoesta J, Rickard M. Age-specific interval breast cancers in New South Wales and meta-analysis of studies of women aged 40-49 years. Journal of medical screening. 2004;11(4):199-206.

422. Collins LC, Connolly JL, Page DL, Goulart RA, Pisano ED, Fajardo LL, et al. Diagnostic agreement in the evaluation of image-guided breast core needle biopsies: results from a randomized clinical trial. American journal of surgical pathology. 2004;28(1):126‐31.

423. Ciatto S, Visioli C, Paci E, Zappa M. Breast density as a determinant of interval cancer at mammographic screening. British journal of cancer. 2004;90(2):393-6.

424. Barton MB, Morley DS, Moore S, Allen JD, Kleinman KP, Emmons KM, et al. Decreasing women's anxieties after abnormal mammograms: a controlled trial. Journal of the National Cancer Institute. 2004;96(7):529‐38.

425. Anderson TJ, Waller M, Ellis IO, Bobrow L, Moss S. Influence of annual mammography from age 40 on breast cancer pathology. Human pathology. 2004;35(10):1252‐9.

426. Vogt TM, Glass A, Glasgow RE, La Chance PA, Lichtenstein E. The safety net: a cost-effective approach to improving breast and cervical cancer screening. Journal of women's health (2002). 2003;12(8):789‐98.

427. Lee SJ, Zelen M. Modelling the early detection of breast cancer. Annals of oncology : official journal of the european society for medical oncology. 2003;14(8):1199‐202.

428. Ciatto S, Rosselli Del Turco M, Burke P, Visioli C, Paci E, Zappa M. Comparison of standard and double reading and computer-aided detection (CAD) of interval cancers at prior negative screening mammograms: blind review. British journal of cancer. 2003;89(9):1645-9.

429. Bjurstam N, Björneld L, Warwick J, Sala E, Duffy SW, Nyström L, et al. The Gothenburg Breast Screening Trial. Cancer. 2003;97(10):2387‐96.

430. Anttinen J, Kuopio T, Nykanen M, Torkkeli H, Saari U, Juhola M. Her-2/neu oncogene amplification and protein over-expression in interval and screen-detected breast cancers. Anticancer research. 2003;23(5b):4213-8.

431. 김준영, 조백현, 허민희, 강성수, 이지현, 이성공, et al. Interval Breast Cancers: Comparisons with Screen Detected Cancers. Annals of Surgical Treatment and Research. 2002;63(6):458-61.

432. Zappa M, Falini P, Bonardi R, Ambrogetti D, Giorgi D, Paci E, et al. Monitoring interval cancers in mammographic screening: the Florence District programme experience. Breast (Edinburgh, Scotland). 2002;11(4):301-5.

433. Thomas DB, Gao DL, Ray RM, Wang WW, Allison CJ, Chen FL, et al. Randomized trial of breast self-examination in Shanghai: final results. Journal of the National Cancer Institute. 2002;94(19):1445‐57.

434. Terry PD, Miller AB, Rohan TE. Obesity and colorectal cancer risk in women. Gut. 2002;51(2):191‐4.

435. Taylor R, Supramaniam R, Rickard M, Estoesta J, Moreira C. Interval breast cancers in New South Wales, Australia, and comparisons with trials and other mammographic screening programmes. Journal of medical screening. 2002;9(1):20‐5.

436. Taplin SH, Rutter CM, Finder C, Mandelson MT, Houn F, White E. Screening mammography: clinical image quality and the risk of interval breast cancer. AJR Am J Roentgenol. 2002;178(4):797-803.

437. Stoddard AM, Fox SA, Costanza ME, Lane DS, Andersen MR, Urban N, et al. Effectiveness of telephone counseling for mammography: results from five randomized trials. Preventive medicine. 2002;34(1):90‐9.

438. Reuben DB, Bassett LW, Hirsch SH, Jackson CA, Bastani R. A randomized clinical trial to assess the benefit of offering on-site mobile mammography in addition to health education for older women. AJR American journal of roentgenology. 2002;179(6):1509‐14.

439. Gower-Thomas K, Fielder HMP, Branston L, Greening S, Beer H, Rogers C. Reviewing interval cancers: time well spent? Clinical radiology. 2002;57(5):384-8.

440. Gao F, Chia K-S, Ng F-C, Ng E-H, Machin D. Interval cancers following breast cancer screening in Singaporean women. International journal of cancer. 2002;101(5):475-9.

441. Crane CEB, Luke CG, Rogers JM, Playford PE, Roder DM. An analysis of factors associated with interval as opposed to screen-detected breast cancers, including hormone therapy and mammographic density. Breast (Edinburgh, Scotland). 2002;11(2):131-6.

442. Champion VL, Skinner CS, Menon U, Seshadri R, Anzalone DC, Rawl SM. Comparisons of tailored mammography interventions at two months postintervention. Annals of behavioral medicine. 2002;24(3):211‐8.

443. Wang H, Bjurstam N, Bjorndal H, Braaten A, Eriksen L, Skaane P, et al. Interval cancers in the Norwegian breast cancer screening program: frequency, characteristics and use of HRT. International journal of cancer. 2001;94(4):594-8.

444. Raja MA, Hubbard A, Salman AR. Interval breast cancer: is it a different type of breast cancer? Breast (Edinburgh, Scotland). 2001;10(2):100-8.

445. Moberg K, Bjurstam N, Wilczek B, Rostgard L, Egge E, Muren C. Computed assisted detection of interval breast cancers. European journal of radiology. 2001;39(2):104-10.

446. McCann J, Britton PD, Warren RM, Hunnam G. Radiological peer review of interval cancers in the East Anglian breast screening programme: what are we missing? East Anglian Breast Screening Programme. Journal of medical screening. 2001;8(2):77-85.

447. Kaas R, Hart AA, Besnard AP, Peterse JL, Rutgers EJ. Impact of mammographic interval on stage and survival after the diagnosis of contralateral breast cancer. The British journal of surgery. 2001;88(1):123-7.

448. Garvican L, Field S. A pilot evaluation of the R2 image checker system and users' response in the detection of interval breast cancers on previous screening films. Clinical radiology. 2001;56(10):833-7.

449. Ganry OF, Peng J, Raverdy NL, Dubreuil AR. Interval cancers in a French breast cancer-screening programme (Somme Department). European journal of cancer prevention : the official journal of the European Cancer Prevention Organisation (ECP). 2001;10(3):269-74.

450. Ellis PM, Butow PN, Tattersall MH, Dunn SM, Houssami N. Randomized clinical trials in oncology: understanding and attitudes predict willingness to participate. Journal of clinical oncology. 2001;19(15):3554‐61.

451. Brown M, Eccles C, Wallis MG. Geographical distribution of breast cancers on the mammogram: an interval cancer database. The British journal of radiology. 2001;74(880):317-22.

452. Britton PD, McCann J, O'Driscoll D, Hunnam G, Warren RM. Interval cancer peer review in East Anglia: implications for monitoring doctors as well as the NHS breast screening programme. Clinical radiology. 2001;56(1):44-9.

453. Allen JD, Stoddard AM, Mays J, Sorensen G. Promoting breast and cervical cancer screening at the workplace: results from the Woman to Woman Study. American journal of public health. 2001;91(4):584‐90.

454. Warren R, Duffy S. Interval cancers as an indicator of performance in breast screening. Breast cancer (Tokyo, Japan). 2000;7(1):9-18.

455. Taplin SH, Barlow WE, Ludman E, MacLehos R, Meyer DM, Seger D, et al. Testing reminder and motivational telephone calls to increase screening mammography: a randomized study. Journal of the National Cancer Institute. 2000;92(3):233‐42.

456. Seger S. Effect of false-positive mammograms on interval breast cancer screening in a health maintenance organization. J Midwifery Womens Health. 2000;45(2):186-7.

457. Morrone D, Giorgi D, Ciatto S, Ceresatto E, Catarzi S, Roselli Del Turco R. Radiologic analysis of interval cancers in the screening program called Florence Woman Project. La Radiologia medica. 2000;100(5):321-5.

458. Miller AB, To T, Baines CJ, Wall C. Canadian National Breast Screening Study-2: 13-year results of a randomized trial in women aged 50-59 years. Journal of the National Cancer Institute. 2000;92(18):1490‐9.

459. Mandelson MT, Oestreicher N, Porter PL, White D, Finder CA, Taplin SH, et al. Breast density as a predictor of mammographic detection: comparison of interval- and screen-detected cancers. Journal of the National Cancer Institute. 2000;92(13):1081-7.

460. Gilliland FD, Joste N, Stauber PM, Hunt WC, Rosenberg R, Redlich G, et al. Biologic characteristics of interval and screen-detected breast cancers. J Natl Cancer Inst. 2000;92(9):743-9.

461. Eisinger F, Juliain-Reynier C, Sobol H. Re: Biologic characteristics of interval and screen-detected breast cancers. Journal of the National Cancer Institute. 2000;92(18):1533-4.

462. de Rijke JM, Schouten LJ, Schreutelkamp JL, Jochem I, Verbeek AL. A blind review and an informed review of interval breast cancer cases in the Limburg screening programme, the Netherlands. J Med Screen. 2000;7(1):19-23.

463. Crane LA, Leakey TA, Ehrsam G, Rimer BK, Warnecke RB. Effectiveness and cost-effectiveness of multiple outcalls to promote mammography among low-income women. Cancer epidemiology, biomarkers & prevention. 2000;9(9):923‐31.

464. Cowan WK, Angus B, Gray JC, Lunt LG, al-Tamimi SR. A study of interval breast cancer within the NHS breast screening programme. Journal of clinical pathology. 2000;53(2):140-6.

465. Amos AF, Kavanagh AM, Cawson J. Radiological review of interval cancers in an Australian mammographic screening programme. Radiology Quality Assurance Group of BreastScreen Victoria. Journal of medical screening. 2000;7(4):184-9.

466. Vitak B, Olsen KE, Månson JC, Arnesson LG, Stål O. Tumour characteristics and survival in patients with invasive interval breast cancer classified according to mammographic findings at the latest screening: a comparison of true interval and missed interval cancers. Eur Radiol. 1999;9(3):460-9.

467. Taylor V, Thompson B, Lessler D, Yasui Y, Montano D, Johnson KM, et al. A clinic-based mammography intervention targeting inner-city women. Journal of general internal medicine. 1999;14(2):104‐11.

468. Tabar L, Vitak B, Chen HH, Prevost TC, Duffy SW. Update of the Swedish two-county trial of breast cancer screening: histologic grade-specific and age-specific results. Swiss surgery. 1999;5(5):199‐204.

469. Porter PL, El-Bastawissi AY, Mandelson MT, Lin MG, Khalid N, Watney EA, et al. Breast tumor characteristics as predictors of mammographic detection: comparison of interval- and screen-detected cancers. Journal of the National Cancer Institute. 1999;91(23):2020-8.

470. Moberg K, Grundstrom H, Tornberg S, Lundquist H, Svane G, Havervall L, et al. Two models for radiological reviewing of interval cancers. Journal of medical screening. 1999;6(1):35-9.

471. Michaelson JS, Halpern E, Kopans DB. Breast cancer: computer simulation method for estimating optimal intervals for screening. Radiology. 1999;212(2):551-60.

472. Liljegren G, Holmberg L, Bergh J, Lindgren A, Tabár L, Nordgren H, et al. 10-Year results after sector resection with or without postoperative radiotherapy for stage I breast cancer: a randomized trial. Journal of clinical oncology. 1999;17(8):2326‐33.

473. Kavanagh AM, Mitchell H, Farrugia H, Giles GG. Monitoring interval cancers in an Australian mammographic screening programme. Journal of medical screening. 1999;6(3):139-43.

474. Exbrayat C, Garnier A, Colonna M, Assouline D, Salicru B, Winckel P, et al. Analysis and classification of interval cancers in a French breast cancer screening programme (departement of Isere). European journal of cancer prevention : the official journal of the European Cancer Prevention Organisation (ECP). 1999;8(3):255-60.

475. Burman ML, Taplin SH, Herta DF, Elmore JG. Effect of false-positive mammograms on interval breast cancer screening in a health maintenance organization. Annals of internal medicine. 1999;131(1):1-6.

476. Vitak B. Invasive interval cancers in the Ostergotland Mammographic Screening Programme: radiological analysis. European radiology. 1998;8(4):639-46.

477. Thurfjell E, Thurfjell MG, Egge E, Bjurstam N. Sensitivity and specificity of computer-assisted breast cancer detection in mammography screening. Acta radiologica (Stockholm, Sweden : 1987). 1998;39(4):384‐8.

478. Rickard MT, Taylor RJ, Fazli MA, El Hassan N. Interval breast cancers in an Australian mammographic screening program. The Medical journal of Australia. 1998;169(4):184-7.

479. Ng EH, Ng FC, Tan PH, Low SC, Chiang G, Tan KP, et al. Results of intermediate measures from a population-based, randomized trial of mammographic screening prevalence and detection of breast carcinoma among Asian women: the Singapore Breast Screening Project. Cancer. 1998;82(8):1521‐8.

480. Moss S, Blanks R. Calculating appropriate target cancer detection rates and expected interval cancer rates for the UK NHS Breast Screening Programme. Interval Cancer Working Group. Journal of epidemiology and community health. 1998;52(2):111-5.

481. de Korvin B, Courtel ML, Bohec C, Durand G, Piette C, Le Freche JN, et al. Radiologic analysis of known-interval cancers after 2 years of organized mass screening for breast cancer in Ille-et-Vilaine. Journal de radiologie. 1998;79(11):1379-86.

482. Bird JA, McPhee SJ, Ha NT, Le B, Davis T, Jenkins CN. Opening pathways to cancer screening for Vietnamese-American women: lay health workers hold a key. Preventive medicine. 1998;27(6):821‐9.

483. Weber BE, Reilly BM. Enhancing mammography use in the inner city. A randomized trial of intensive case management. Archives of internal medicine. 1997;157(20):2345‐9.

484. Vitak B, Stal O, Manson JC, Thomas BA, Arnesson LG, Ekelund L, et al. Interval cancers and cancers in non-attenders in the Ostergotland Mammographic Screening Programme. Duration between screening and diagnosis, S-phase fraction and distant recurrence. European journal of cancer (Oxford, England : 1990). 1997;33(9):1453-60.

485. Sylvester PA, Vipond MN, Kutt E, Davies JD, Webb AJ, Farndon JR. A comparative audit of prevalent, incident and interval cancers in the Avon breast screening programme. Annals of the Royal College of Surgeons of England. 1997;79(4):272-5.

486. Sylvester PA, Kutt E, Baird A, Vipond MN, Webb AJ, Farndon JR. Rate and classification of interval cancers in the breast screening programme. Annals of the Royal College of Surgeons of England. 1997;79(4):276-7.

487. Paci E, Alexander FE. Study design of randomized controlled clinical trials of breast cancer screening. Journal of the National Cancer Institute Monographs. 1997(22):21‐5.

488. Miller AB, To T, Baines CJ, Wall C. The Canadian National Breast Screening Study: update on breast cancer mortality. Journal of the National Cancer Institute Monographs. 1997(22):37‐41.

489. Lenner P, Jonsson H. Excess mortality from breast cancer in relation to mammography screening in northern Sweden. Journal of medical screening. 1997;4(1):6‐9.

490. Larsson LG, Andersson I, Bjurstam N, Fagerberg G, Frisell J, Tabár L, et al. Updated overview of the Swedish Randomized Trials on Breast Cancer Screening with Mammography: age group 40-49 at randomization. Journal of the National Cancer Institute Monographs. 1997(22):57‐61.

491. Klemi PJ, Toikkanen S, Rasanen O, Parvinen I, Joensuu H. Mammography screening interval and the frequency of interval cancers in a population-based screening. British journal of cancer. 1997;75(5):762-6.

492. Hendrick RE, Smith RA, Rutledge JH, Smart CR. Benefit of screening mammography in women aged 40-49: a new meta-analysis of randomized controlled trials. Journal of the National Cancer Institute Monographs. 1997(22):87‐92.

493. Hakama M, Pukkala E, Heikkilä M, Kallio M. Effectiveness of the public health policy for breast cancer screening in Finland: population based cohort study. BMJ (Clinical research ed). 1997;314(7084):864‐7.

494. Frisell J, Lidbrink E, Hellström L, Rutqvist LE. Followup after 11 years--update of mortality results in the Stockholm mammographic screening trial. Breast cancer research and treatment. 1997;45(3):263‐70.

495. Frisell J, Lidbrink E. The Stockholm Mammographic Screening Trial: risks and benefits in age group 40-49 years. Journal of the National Cancer Institute Monographs. 1997(22):49‐51.

496. Boyd NF, Greenberg C, Lockwood G, Little L, Martin L, Byng J, et al. Effects at two years of a low-fat, high-carbohydrate diet on radiologic features of the breast: results from a randomized trial. Canadian Diet and Breast Cancer Prevention Study Group. Journal of the National Cancer Institute. 1997;89(7):488‐96.

497. Bjurstam N, Björneld L, Duffy SW, Smith TC, Cahlin E, Eriksson O, et al. The Gothenburg breast screening trial: first results on mortality, incidence, and mode of detection for women ages 39-49 years at randomization. Cancer. 1997;80(11):2091‐9.

498. Vizcaino Esteve I, Vilar Samper J, Ruiz Perales F. Analysis of interval cancers in the Program of Early Diagnosis of Cancer of the Breast of the Community of Valencia. Group of Readers of the Prevention Units of Cancer of the Breast. Revista clinica espanola. 1996;196(10):703-5.

499. Burrell HC, Sibbering DM, Wilson AR, Pinder SE, Evans AJ, Yeoman LJ, et al. Screening interval breast cancers: mammographic features and prognosis factors. Radiology. 1996;199(3):811-7.

500. Beaulieu MD, Béland F, Roy D, Falardeau M, Hébert G. Factors determining compliance with screening mammography. CMAJ : Canadian Medical Association journal. 1996;154(9):1335‐43.

501. Asbury D, Boggis CR, Sheals D, Threlfall AG, Woodman CB. NHS breast screening programme: is the high incidence of interval cancers inevitable? BMJ (Clinical research ed). 1996;313(7069):1369-70.

502. Woodman CB, Threlfall AG, Boggis CR, Prior P. Is the three year breast screening interval too long? Occurrence of interval cancers in NHS breast screening programme's north western region. BMJ (Clinical research ed). 1995;310(6974):224-6.

503. Wald NJ, Murphy P, Major P, Parkes C, Townsend J, Frost C. UKCCCR multicentre randomised controlled trial of one and two view mammography in breast cancer screening. BMJ (Clinical research ed). 1995;311(7014):1189‐93.

504. Schaffer P, Renaud R, Gairard B, Guldenfels C. Breast cancer between 2 screenings: is a 3-year interval too long? Revue d'epidemiologie et de sante publique. 1995;43(3):290-3.

505. Kopans DB. Re: Interval breast cancers in the screening mammography program of British Columbia: analysis and classification. AJR American journal of roentgenology. 1995;164(5):1298-9.

506. Kerlikowske K, Grady D, Rubin SM, Sandrock C, Ernster VL. Efficacy of screening mammography. A meta-analysis. JAMA. 1995;273(2):149‐54.

507. Goff JM, Molloy M, Debbas MT, Hale DA, Jaques DP. Long-term impact of previous breast biopsy on breast cancer screening modalities. J Surg Oncol. 1995;59(1):18-20.

508. Field S, Michell MJ, Wallis MG, Wilson AR. What should be done about interval breast cancers? BMJ (Clinical research ed). 1995;310(6974):203-4.

509. Duncan AA, Wallis MG. Classifying interval cancers. Clinical radiology. 1995;50(11):774-7.

510. Day N, McCann J, Camilleri-Ferrante C, Britton P, Hurst G, Cush S, et al. Monitoring interval cancers in breast screening programmes: the east Anglian experience. Quality Assurance Management Group of the East Anglian Breast Screening Programme. Journal of medical screening. 1995;2(4):180-5.

511. Brekelmans CT, Peeters PH, Deurenberg JJ, Collette HJ. Survival in interval breast cancer in the DOM screening programme. Eur J Cancer. 1995;31a(11):1830-5.

512. Boer R, De Koning HJ, van Oortmarssen GJ, van der Maas PJ. Screening for breast cancer. Incidence of interval cancer and detection rate of first screenings are inconsistent. BMJ (Clinical research ed). 1995;310(6985):1002-.

513. Spiegelman D, Colditz GA, Hunter D, Hertzmark E. Validation of the Gail et al. model for predicting individual breast cancer risk. Journal of the National Cancer Institute. 1994;86(8):600‐7.

514. Meldrum P, Turnbull D, Dobson HM, Colquhoun C, Gilmour WH, McIlwaine GM. Tailored written invitations for second round breast cancer screening: a randomised controlled trial. Journal of medical screening. 1994;1(4):245‐8.

515. Liljegren G, Holmberg L, Adami HO, Westman G, Graffman S, Bergh J. Sector resection with or without postoperative radiotherapy for stage I breast cancer: five-year results of a randomized trial. Uppsala-Orebro Breast Cancer Study Group. Journal of the National Cancer Institute. 1994;86(9):717‐22.

516. Koivunen D, Zhang X, Blackwell C, Adelstein E, Humphrey L. Interval breast cancers are not biologically distinct--just more difficult to diagnose. Am J Surg. 1994;168(6):538-42.

517. Kiefe CI, McKay SV, Halevy A, Brody BA. Is cost a barrier to screening mammography for low-income women receiving Medicare benefits? A randomized trial. Archives of internal medicine. 1994;154(11):1217‐24.

518. Frischbier HJ. Controversial attitude to mammography screening in asymptomatic women between 40 and 50 years of age. Geburtshilfe und Frauenheilkunde. 1994;54(1):1‐11.

519. Burhenne HJ, Burhenne LW, Goldberg F, Hislop TG, Worth AJ, Rebbeck PM, et al. Interval breast cancers in the Screening Mammography Program of British Columbia: analysis and classification. AJR American journal of roentgenology. 1994;162(5):1067-71; discussion 72-5.

520. Brekelmans CT, Peeters PH, Faber JA, Deurenberg JJ, Collette HJ. The epidemiological profile of women with an interval cancer in the DOM screening programme. Breast Cancer Res Treat. 1994;30(3):223-32.

521. Baines CJ. The Canadian National Breast Screening Study: a perspective on criticisms. Annals of internal medicine. 1994;120(4):326‐34.

522. Arnesson LG, Smeds S, Fagerberg G. Recurrence-free survival in patients with small breast cancer. An analysis of cancers 10 mm or less detected clinically and by screening. European journal of surgery = Acta chirurgica. 1994;160(5):271‐6.

523. Watmough DJ. Interval breast cancers. AJR American journal of roentgenology. 1993;161(1):207-8.

524. Vandenbroucke A, Bourdon C. Breast cancer screening programmes--results of studies in foreign countries--situation in Belgium. European journal of cancer prevention. 1993;2(3):269‐74.

525. van Dijck JA, Verbeek AL, Hendriks JH, Holland R. The current detectability of breast cancer in a mammographic screening program. A review of the previous mammograms of interval and screen-detected cancers. Cancer. 1993;72(6):1933-8.

526. Tabar L, Duffy SW, Burhenne LW. New Swedish breast cancer detection results for women aged 40-49. Cancer. 1993;72(4 Suppl):1437‐48.

527. Nyström L, Rutqvist LE, Wall S, Lindgren A, Lindqvist M, Rydén S, et al. Breast cancer screening with mammography: overview of Swedish randomised trials. Lancet (london, england). 1993;341(8851):973‐8.

528. Moss SM, Coleman DA, Ellman R, Chamberlain J, Forrest AP, Kirkpatrick AE, et al. Interval cancers and sensitivity in the screening centres of the UK trial of early detection of breast cancer. European journal of cancer (Oxford, England : 1990). 1993;29A(2):255-8.

529. Holowaty PH, Miller AB, Baines CJ, Risch H. Canadian National Breast Screening Study: first screen results as predictors of future breast cancer risk. Cancer epidemiology, biomarkers & prevention. 1993;2(1):11‐9.

530. Fletcher SW, Harris RP, Gonzalez JJ, Degnan D, Lannin DR, Strecher VJ, et al. Increasing mammography utilization: a controlled study. Journal of the National Cancer Institute. 1993;85(2):112‐20.

531. Miller AB, Baines CJ, To T, Wall C. Canadian National Breast Screening Study: 1. Breast cancer detection and death rates among women aged 40 to 49 years. CMAJ : Canadian Medical Association journal. 1992;147(10):1459‐76.

532. Frisell J, von Rosen A, Wiege M, Nilsson B, Goldman S. Interval cancer and survival in a randomized breast cancer screening trial in Stockholm. Breast cancer research and treatment. 1992;24(1):11‐6.

533. Frisell J, Eklund G, Hellström L, Lidbrink E, Rutqvist LE, Somell A. Randomized study of mammography screening--preliminary report on mortality in the Stockholm trial. Breast cancer research and treatment. 1991;18(1):49‐56.

534. Cuckle H. Breast cancer screening by mammography: an overview. Clinical radiology. 1991;43(2):77‐80.

535. Peeters PH, Verbeek AL, Zielhuis GA, Vooijs GP, Hendriks JH, Mravunac M. Breast cancer screening in women over age 50. A critical appraisal. Acta Radiol. 1990;31(3):225-31.

536. Baines CJ, McFarlane DV, Miller AB. The role of the reference radiologist. Estimates of inter-observer agreement and potential delay in cancer detection in the national breast screening study. Invest Radiol. 1990;25(9):971-6.

537. Peeters PH, Verbeek AL, Hendriks JH, Holland R, Mravunac M, Vooijs GP. The occurrence of interval cancers in the Nijmegen screening programme. British journal of cancer. 1989;59(6):929-32.

538. Lewars MD. Reviewing interval cancers: time well spent? Clinical radiology. 2002;57(10):955-; author reply

539. Narod SA, Dube MP. Re: Biologic characteristics of interval and screen-detected breast cancers. Journal of the National Cancer Institute. 2001;93(2):151-2.

540. Pulido-Carmona C, Romero-Martin S, Raya-Povedano JL, Cara-Garcia M, Font-Ugalde P, Elias-Cabot E, et al. Interval cancer in the Córdoba Breast Tomosynthesis Screening Trial (CBTST): comparison of digital breast tomosynthesis plus digital mammography to digital mammography alone. European Radiology. 2024.

541. Pattacini P, Nitrosi A, Giorgi Rossi P, Duffy SW, Iotti V, Ginocchi V, et al. A Randomized Trial Comparing Breast Cancer Incidence and Interval Cancers after Tomosynthesis Plus Mammography versus Mammography Alone. Radiology. 2022;303(2):256‐66.

542. Armaroli P, Frigerio A, Correale L, Ponti A, Artuso F, Casella D, et al. A randomised controlled trial of digital breast tomosynthesis vs digital mammography as primary screening tests: screening results over subsequent episodes of the Proteus Donna study. International journal of cancer. 2022;151(10):1778‐90.

543. Hofvind S, Moshina N, Holen Å, Danielsen AS, Lee CI, Houssami N, et al. Interval and Subsequent Round Breast Cancer in a Randomized Controlled Trial Comparing Digital Breast Tomosynthesis and Digital Mammography Screening. Radiology. 2021;300(1):66‐76.

544. Winter AM, Kazmi S, Hardy AK, Bennett DL. Comparison of interval breast cancers with 2D digital mammography versus 3D digital breast tomosynthesis in a large community-based practice. Breast J. 2020;26(10):1953-9.

545. Hovda T, Holen AS, Lang K, Albertsen JL, Bjorndal H, Brandal SHB, et al. Interval and Consecutive Round Breast Cancer after Digital Breast Tomosynthesis and Synthetic 2D Mammography versus Standard 2D Digital Mammography in BreastScreen Norway. Radiology. 2020;294(2):256-64.

546. Bernardi D, Gentilini MA, De Nisi M, Pellegrini M, Fanto C, Valentini M, et al. Effect of implementing digital breast tomosynthesis (DBT) instead of mammography on population screening outcomes including interval cancer rates: Results of the Trento DBT pilot evaluation. Breast. 2020;50:135-40.

547. Hovda T, Brandal SHB, Sebuødegård S, Holen Å S, Bjørndal H, Skaane P, et al. Screening outcome for consecutive examinations with digital breast tomosynthesis versus standard digital mammography in a population-based screening program. Eur Radiol. 2019;29(12):6991-9.

548. Skaane P, Sebuødegård S, Bandos AI, Gur D, Østerås BH, Gullien R, et al. Performance of breast cancer screening using digital breast tomosynthesis: results from the prospective population-based Oslo Tomosynthesis Screening Trial. Breast Cancer Res Treat. 2018;169(3):489-96.

549. Houssami N, Bernardi D, Caumo F, Brunelli S, Fantò C, Valentini M, et al. Interval breast cancers in the 'screening with tomosynthesis or standard mammography' (STORM) population-based trial. Breast. 2018;38:150-3.

550. McDonald ES, Oustimov A, Weinstein SP, Synnestvedt MB, Schnall M, Conant EF. Effectiveness of Digital Breast Tomosynthesis Compared With Digital Mammography: Outcomes Analysis From 3 Years of Breast Cancer Screening. JAMA Oncol. 2016;2(6):737-43.
