# Supplementary material for: Impact of digital breast tomosynthesis on screening performance and interval cancer rates compared to digital mammography: A meta-analysis
Source: PLoS One. 2025 Jan 31;20(1):e0315466. doi: 10.1371/journal.pone.0315466 (PMC11785311; doi:10.1371/journal.pone.0315466)
Supplement: S4 Table — (DOCX) [file pone.0315466.s006.docx]

**S4 Table. Original study data.**

| **Study** | **TP** | **FP** | **FN** | **TN** | **Index Test** |
| --- | --- | --- | --- | --- | --- |
| Hofvind 2021 | 95 | 349 | 20 | 13917 | DBT |
|  | 87 | 484 | 29 | 13773 | DM |
| Hovda 2020 | 324 | 834 | 68 | 33415 | SM/DBT |
|  | 355 | 1518 | 88 | 55802 | DM |
| Pattacini 2022 | 101 | 410 | 21 | 12824 | DM/DBT |
|  | 61 | 461 | 22 | 12977 | DM |
| Pulido-Carmona 2024 | 98 | 1098 | 15 | 14872 | DM/DBT |
|  | 146 | 1225 | 43 | 22416 | DM |
| Armaroli 2022 | 256 | 1699 | 42 | 28847 | DBT |
|  | 248 | 1943 | 64 | 40767 | DM |
| Bernardi 2021 | 402 | 778 | 51 | 45112 | SM/DBT |
|  | 205 | 996 | 51 | 36184 | DM |
| Houssami 2018 | 59 | 254 | 9 | 6970 | DM/DBT |
|  | 133 | 322 | 40 | 24563 | DM |
| Skaane 2018 | 227 | 593 | 51 | 23427 | DM/DBT |
|  | 378 | 2152 | 118 | 57229 | DM |
| McDonald 2016 | 196 | 2842 | 17 | 30685 | DM/DBT |
|  | 49 | 1067 | 8 | 9604 | DM |
| Hovda 2019 | 44 | 119 | 26 | 8391 | DM/DBT |
|  | 48 | 323 | 23 | 10108 | DM |
| Winter 2020 | 709 | 9638 | 40 | 109359 | DBT |
|  | 411 | 12097 | 35 | 104556 | DM |

TP=true positives, FP=false positives, FN=false negatives (interval breast cancer), TN=true negatives. Index Test: DBT, DM/DBT, SM/DBT, DM.
