# Supplementary figures and images for: Impact of digital breast tomosynthesis on screening performance and interval cancer rates compared to digital mammography: A meta-analysis
Source: PLoS One. 2025 Jan 31;20(1):e0315466. doi: 10.1371/journal.pone.0315466 (PMC11785311; doi:10.1371/journal.pone.0315466)

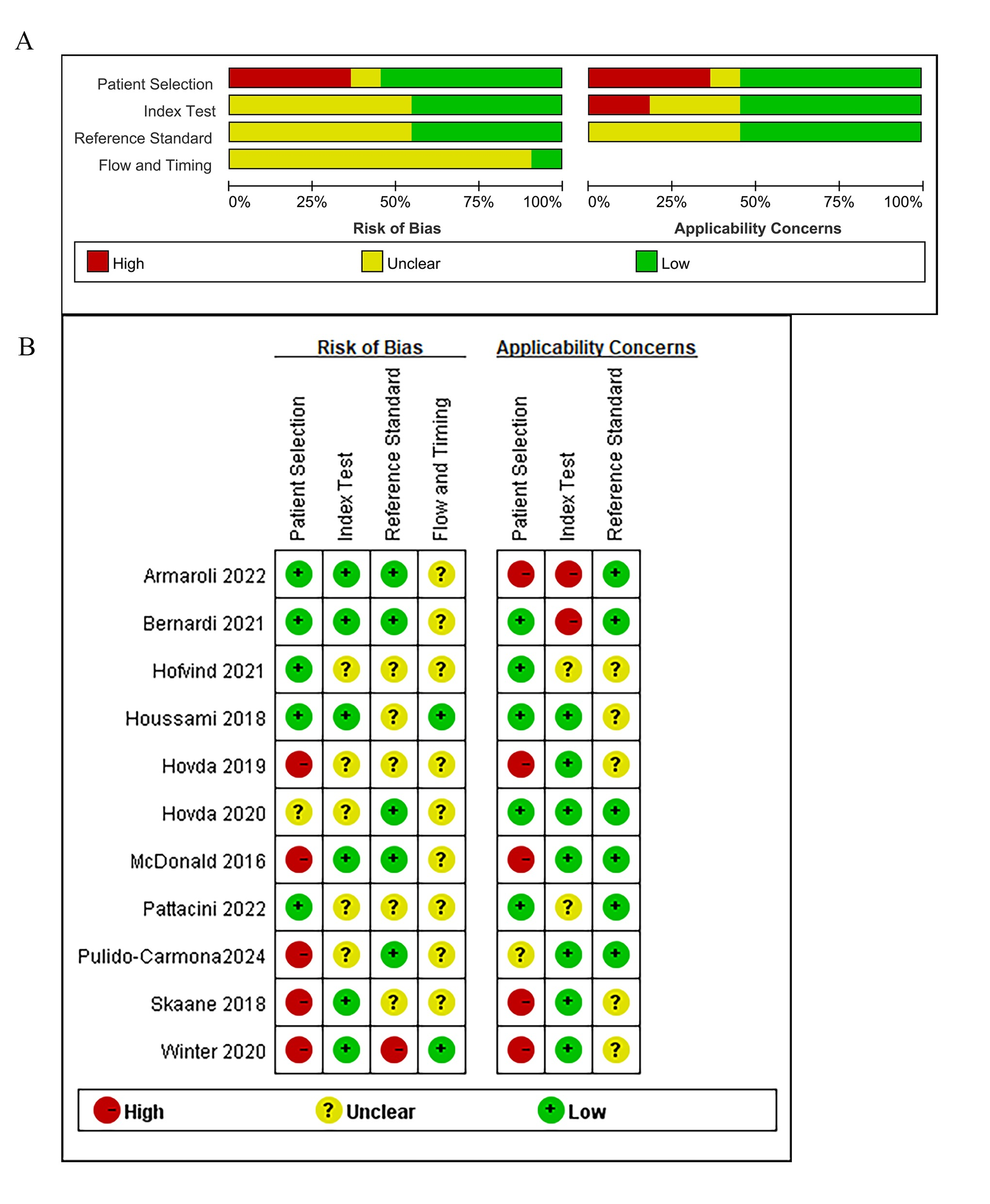

Supplement: S1 Fig — (A)Risk of bias and applicability concerns graph, (B)Risk of bias and applicability concerns summary. (TIF) [file pone.0315466.s007.tif]
