# Supplementary material for: Impact of digital breast tomosynthesis on screening performance and interval cancer rates compared to digital mammography: A meta-analysis
Source: PLoS One. 2025 Jan 31;20(1):e0315466. doi: 10.1371/journal.pone.0315466 (PMC11785311; doi:10.1371/journal.pone.0315466)
Supplement: Attachment — Submitted filename: reply.docx [file pone.0315466.s008.docx]

August 30, 2024

Dear Kate-Lyn Maaño Claro,

Thank you very much for your response and valuable suggestions.

We have re-uploaded the manuscript in accordance with PRISMA guidelines. The completed PRISMA checklist has been uploaded as supporting information in a file named 'S2 File PRISMA checklist'. Additionally, a flowchart has been included as Figure 1 and saved in a file named 'Fig 1 Flowchart'.

We would like to clarify that the data for our meta-analysis consist of studies included in a literature database, selected based on predefined criteria. A meta-analysis utilizes statistical techniques within a systematic review to synthesize the findings of the included studies. Therefore, ethical approval is not required for this type of research.

We hope that the revised manuscript will fulfill the requirements for submission. We appreciate your consideration of our manuscript and look forward to receiving the reviewers' comments. Should you have any questions, please do not hesitate to contact me at the address provided below.

Thank you and best regards.

Yours Sincerely,

Juan Yao

E-mail: 13139929005@163.com
